# Deuxième Congrès de la Soguipit « Pathologies infectieuses émergentes et ré-émergentes en Afrique : gouvernance, Défis et perspectives » 13 - 14 Octobre 2022, Conakry, Guinée

**DOI:** 10.48327/mtsi.v3i2.2023.393

**Published:** 2023-06-29

**Authors:** Mamadou Saliou SOW, Alice DESCLAUX, Alpha Kabinet KEITA, Abdoulaye MAKANERA, Mamadou Abdoulaye TRAORE, Abdoulaye TRAORE, Abdoulaye TOURE, Michel SAGNO, Moustapha DIOP, Abdoulaye Oury BARRY, Mamadou Oury Safiatou DIALLO, Alioune CAMARA, Alexandre DELAMOU, Frederic LE MARCIS, Louise FORTES DENGUENOVO, Armel PODA, Aboubacar ALHASSANE, Boushab MOHAMED, Mamoudou SAVADOGO, Alphonse TOLNO, Dembo DIAKITE, Mamadou Oury KEITA, Yacouba CISSOKO

**Affiliations:** Service des Maladies Infectieuses, Hôpital national Donka, CHU Conakry, BP: 234, Conakry, Guinée

**Keywords:** Épidémies, Marburg, Ébola, SARS-CoV-2, Dengue, Covid-19, VIH, Abcès, Infections, ARV, Influenza aviaire, Hépatite, Paludisme, HAE, Maladie rénale, Tétanos, Behçet, Tuberculose, IPP, Hémoglobine N-Baltimore, Méningites, Déficience visuelle, Afrique subsaharienne, Epidemics, Marburg, Ebola, SARS-CoV-2, Dengue, Covid-19, HIV, Abscesses, Infections, ARV, Avian influenza, Hepatitis, Malaria, HAE, Kidney disease, Tetanus, Behçet, Tuberculosis, PPI, Haemoglobin N-Baltimore, Meningitis, Visual impairment, Sub-Saharan Africa

Le deuxième congrès de la Société guinéenne de pathologie infectieuse et tropicale (SOGUIPIT) intitulé « Pathologies infectieuses émergentes et ré-émergentes en Afrique : Gouvernance, défis et perspectives » s'est déroulé du 13 au 14 octobre 2022. Environ 300 personnes venant de 10 pays étaient présentes physiquement ou par visioconférence.

La conférence introductive a été présentée par le président de la SOGUIPIT, le Professeur Mamadou Saliou Sow, sous le thème « Impact des épidémies sur la continuité des soins en Guinée ». Dans son allocution, il a rappelé l'historique des principales épidémies survenues depuis la création du Service des maladies infectieuses appelé initialement Service des maladies diarrhéiques à cause de la première épidémie de choléra en 1970, en insistant sur les fièvres hémorragiques (Ébola, Marburg, Lassa) survenues au cours des cinq dernières années. Il a aussi évoqué ce qu'il a appelé les maladies du quotidien (VIH et infections opportunistes, tuberculose, méningite, paludisme, bactéries multirésistantes, envenimations…) qui tuent en silence. Il a fait une revue de la littérature sur l'impact des épidémies sur la continuité des soins à travers le monde et particulièrement en Guinée, et il a appelé les autorités à mobiliser des ressources financières et humaines (ouverture de formations diplomantes de courte et longue durée) afin de faire face aux nouvelles maladies émergentes et ré-émergentes.

Dans une visioconférence sur la gestion des épidémies, le Professeur Didier Raoult a évoqué les stratégies utilisées par l'IHU-Méditerranée Infection de Marseille dans la lutte contre la Covid-19. Il a souligné que l'hypoxie n'est pas associée à une hypercapnie, d'où l'intérêt de donner de l'oxygène très tôt aux patients atteints de la Covid-19. Les corticoïdes à faibles doses entraînent une amélioration en phase précoce, de la Covid-19 et les vitamines D et C apportent une amélioration dans 65 % des cas. Enfin, il a reconnu que l'hydroxychloroquine n'est pas parfaite pour le traitement de la Covid-19. Durant cette pandémie environ 47 milliards de dollars ont été investis, ce qui n'avait jamais été réalisé dans l'histoire des pandémies.

Au cours des sessions parallèles, 29 présentations orales ont concerné les fièvres hémorragiques virales (aspects cliniques, diagnostiques biologiques dont la sérologie, la biologie moléculaire, les avancées thérapeutiques et vaccinales mais aussi les aspects socio-anthropologiques). Ces communications ont souligné l'importance de la recherche des mutations dans la surveillance des maladies infectieuses.

**Première journée du congrès, jeudi 13 octobre 2022**

La cérémonie d'ouverture a commencé vers 15 h en présence du Ministre de la Santé et de l'hygiène publique avec les discours successifs du président de la SOGUIPIT, du représentant de l'OMS en Guinée et du Ministre de la Santé.

Dans son allocution, ce dernier a souhaité la bienvenue à nos illustres invités et a remercié les partenaires publics et privés pour leur soutien à la SOGUIPIT. En évoquant la situation sanitaire de la Guinée depuis sa prise de fonction, il a parlé de la gestion successive et simultanée des maladies émergentes parmi lesquelles Ébola, Marburg, Lassa sur fond d'une pandémie à SARS-CoV-2. Il a donc réitéré, au nom du Président le Colonel Mamady Doumbouya et de son Premier ministre M. Bernard Gomou, chef du gouvernement, son engagement à accompagner la SOGUIPIT pour mener à bien ses activités. Avant de déclarer ouvert ce deuxième congrès, il a invité les participants étrangers à visiter l'intérieur du pays. La cérémonie s'est terminée par une note musicale mettant en valeur la culture guinéenne.

La session de l'après-midi a commencé par une conférence du Conseiller régional de l'OMS en gestion des risques sanitaires et préparation des pays, basé à Dakar, intitulée « Préparation et réponse aux urgences de santé publique en Afrique de l'Ouest et centrale ». Le Docteur Amadou Bailo Diallo a évoqué les progrès enregistrés, les défis à relever, les perspectives de la préparation et la réponse aux urgences de santé publique en Afrique de l'Ouest et centrale. L’équipe du Dr Diallo a pu installer en Afrique 53 laboratoires de séquençage.

Un symposium animé par LABONET a abordé le thème de la place du laboratoire dans la lutte contre les infections.

La société TULIP Industrie a ensuite fait une présentation intitulée « La Guinée au top de la technologie télémédecine ».

La conférence du Professeur Loïc Epelboin portait sur « La fièvre Q : une future zoonose émergente en Afrique ? ». Il a décrit les aspects cliniques, épidémiologiques, thérapeutiques ainsi que la répartition de la fièvre Q en Afrique. Il a invité les participants à s'associer pour la rédaction d'une revue scientifique sur la fièvre Q, mais aussi à initier de projets de recherche sur cette pathologie méconnue en Afrique et particulièrement en Guinée que la SOGUIPIT appelle également de ses vœux afin d’établir un état de lieu de la fièvre Q en Afrique.

La deuxième conférence a été présentée par le Professeur Fodé Abass Cissé sur « L'impact de la Covid-19 sur la prise en charge des AVC ».

Les sessions de l'après-midi ont concerné la Covid-19 et les maladies chroniques transmissibles (VIH, TB) ou non transmissibles.

**Deuxième journée du congrès, vendredi 14 octobre 2022**

Au cours de la 2^e^ journée du congrès, le Professeur Naby Moussa Balde, directeur national de l’épidémiologie et de la lutte contre les maladies au Ministère de la Santé, a présenté une communication sur le diabète et la Covid-19. Il a évoqué la vulnérabilité des patients diabétiques atteints de Covid-19. Il a également souligné l'importance à accorder aux maladies non transmissibles pendant les épidémies.

Avec sa deuxième conférence intitulée « L'histoplasmose : infection opportuniste ignorée en Afrique de l'Ouest et centrale ? », le Professeur Loïc Epelboin sur a abordé les aspects cliniques, microbiologiques et thérapeutiques. Il a souligné la nécessité de rechercher cette infection chez les patients atteints du VIH.

Au cours de 2 sessions parallèles, 23 présentations orales ont traité, notamment, du VIH, de la tuberculose, des fièvres typhoïdes et de l'hépatite B…

**La séance de posters a permis d'afficher 54 communications.**

Un panel intitulé « Les épidémies multiples et simultanées en Guinée : Problématique de la prise en charge, défis et perspectives » a été modéré par le Professeur Fodé Bangaly Sako, directeur adjoint national de l’épidémiologie et de la lutte contre les maladies au Ministère de la Santé. Ont pris part à ce panel, des représentants de l'Agence nationale de sécurité sanitaire (ANSS), de l'OMS, de la Faculté des sciences et techniques de la santé, de la chaire de Santé publique, et des laboratoires biomédicaux de l'Institut national de santé publique. Plusieurs aspects ont été abordés, en particulier la prise en charge pluridisciplinaire (démarche clinique concernant les personnes vulnérables, les diabétiques, les hypertendus…), la biologie médicale, la vaccination, les engagements communautaires et la mobilisation des ressources humaines et financières. Il a été fortement recommandé au cours de ce panel la création de deux diplômes afin d'améliorer la prise en charge des pathologies émergentes et ré-émergentes:

un diplôme de courte durée (de type DU) sur la prise en charge des maladies émergentes et ré-émergentes avec à moyen terme la création d'un Centre d'application; un diplôme de longue durée (de type DES) en maladies infectieuses.

Les autorités compétentes se sont engagées à assister la SOGUIPIT et recommandent aux partenaires techniques et financiers d'appuyer la mise en place de ces formations.

Après lecture du rapport de synthèse de ces deux jours en présence des représentants de l'OMS en Guinée, du Ministère de la Santé, du directeur de l'UAGCP, du président de la SOGUIPIT et du représentant de la Société de pathologie infectieuse de langue française (SPILF), la clôture a été prononcée par le Directeur général de l'ANSS, le Professeur Fodé Amara Traoré.

Une troupe culturelle de Guinée a animé la soirée autour d'un repas de socialisation avec remise des trophées aux principaux sponsors et aux trois plus jeunes chercheurs de moins de 28 ans qui ont présenté les meilleures communications.

**Figure 1:**
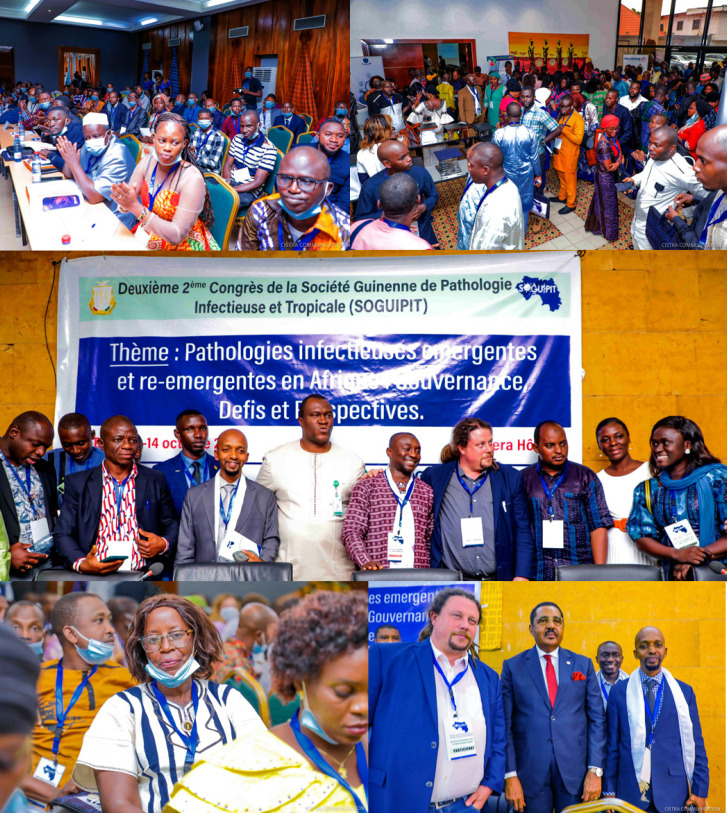
Deuxième congrès de la SOGUIPIT « Pathologies infectieuses émergentes et ré-émergentes en Afrique : Gouvernance, défis et perspectives » 13 - 14 octobre 2022, Conakry, Guinée. En bas à droite, Mamadou Péthé Diallo, Ministre de la santé et de l'hygiène publique, entre Loïc Epelboin (UMIT CF Cayenne, Guyane, France) et Mamadou Saliou Sow (Président de la SOGUIPIT), derrière, Fodé Banaly Sako, Directeur général adjoint de la Direction nationale de l’épidémiologie et de la lutte contre la maladie, Ministère de la santé et trésorier de la SOGUIPIT (crédit photo : Cistra Communication) Second SOGUIPIT Congress “Emerging and re-emerging infectious diseases in Africa: Governance, challenges and prospects”. 13 - 14 October 2022, Conakry, Guinea. Bottom right, Mamadou Péthé Diallo, Minister of Health and Public Hygiene, between Loïc Epelboin (UMIT CF Cayenne, French Guiana, France) and Mamadou Saliou Sow (Chairman of SOGUIPIT), in the background, Fodé Banaly Sako, Deputy Director General of the National Epidemiology and Disease Control Directorate, Ministry of Health and Treasurer of SOGUIPIT (photo credit: Cistra Communication)

