# Supplementary material for: Second Soguipit Congress “emerging and re-emerging infectious diseases in Africa: governance, challenges and prospects”. 13 - 14 October 2022, Conakry, Guinea
Source: Med Trop Sante Int. 2023 Jun 29;3(2):mtsi.v3i2.2023.393. [Article in French] doi: 10.48327/mtsi.v3i2.2023.393 (PMC10387312; doi:10.48327/mtsi.v3i2.2023.393)
Supplement: Related texts

# Research article: Recherche du virus de Marburg chez les chauves-souris en Guinée

**Published:** 2023-06-29

**Authors:** Aminata MBAYE, Abdoul SOUMAH, Joel Balle KOIVOGUI, Jean Louis MONEMOU, Thierno Amadou BALDE, Moriba Kowa POVOGUI, Abdoulaye TOURÉ, Alpha Kabinet KEITA

**Affiliations:** 1 Centre de recherche et de formation en infectiologie de Guinée (CERFIG), Campus Hadja Mafory Bangoura, Conakry, Guinée; 2 Institut de recherche pour le développement (IRD), Montpellier, France

La république de Guinée est le théâtre d'infections virales hémorragiques ces dernières années. En effet, après l’épidémie d’Ébola, un cas confirmé de Marburg chez un patient habitant à Temessadou M'Boké, un village de la préfecture de Guéckédou en Guinée a été détecté. Ce patient avait des activités dans une grotte où l'on trouve fréquemment des chauves-souris. Le virus de Marburg appartient à la même famille (Filoviridae) que le virus Ébola et les chauves-souris sont généralement signalées comme porteuses du virus. L'espèce *Rousettus aegyptiacus* est désignée comme l'hôte naturel du virus de Marburg. L'objectif de cette étude est de voir la présence du virus dans la population de chauves-souris circulant en Guinée. La capture des chauves-souris a eu lieu dans sept villages de la préfecture de Guéckédou en région forestière de Guinée. Les échantillons ont été prélevés sur différents tissus (cœur, foie, intestin, poumon, rate, rein, testicule) de chauves-souris et envoyés au Centre de recherche et de formation en infectiologie de Guinée (CERFIG) pour analyse biologique. L'ARN a été extrait à l'aide du kit NucliSens miniMag. Après une réaction de transcrip-tase inverse, le protocole de PCR nichée pour le virus de Marburg a été réalisé. Au total, 27 chauves-souris appartenant à trois familles (Pteropodidae, Rhinolophidae et Hipposide-ridae) ont été collectées. Les espèces *Rousettus aegyptiacus* (14), *Lissonycteris angolensis* (4), *Hypsignathus monstrosus* (1), *Hipposideros ruber* (6), *Rhinolophus fumigatus* (1) ont été trouvées.

La chauve-souris *Rousettus aegyptiacus* est présente dans 5 villages sur un total de 7 villages. Tous les échantillons ont été testés négatifs pour Marburg. Cette étude a montré la présence de chauves-souris comme *Rousettus aegyptiacus* à Guéckédou. Une étude avec davantage d’échantillons et utilisant les technologies récentes de la biologie moléculaire est donc nécessaire.

# Research article: Preuve sérologique de l'infection par le virus Ébola dans les zones rurales de la Guinée avant l’épidémie ouest-africaine de 2014

**Published:** 2023-06-29

**Authors:** Abdoulaye TOURÉ, Alpha K. KEITA, Haby DIALLO, Abdoul Karim SOUMAH, Thibaut Armel Cherif GNIMADI, Christelle BUTEL, Guillaume THAURIGNAC, Martine PEETERS, Éric DELAPORTE, Ahidjo AYOUBA

**Affiliations:** 1 Centre de recherche et de formation en infectiologie de Guinée (CERFIG), Conakry, Guinée; 2 Institut de recherche pour le développement, IRD/ UMI233/INSERM-U1175, Université de Montpellier, Montpellier, France

## Contexte

Depuis 1976, plusieurs dizaines d’épidémies de virus Ébola (EBOV) ont été signalées chez l'homme en Afrique. Depuis 1994, leur fréquence a augmenté et les études de modélisation suggèrent que les régions les plus à risque d'une future épidémie d’Ébola englobent 22 pays d'Afrique centrale et occidentale, y compris la Guinée. L'objectif de cette étude était de rechercher la preuve d'une circulation de l'EBOV en Guinée avant 2014.

## Matériel et méthodes

L’étude a porté sur les échantillons de sang collectés dans le cadre de l'Enquête démographique et de santé (EDS) 2012 menée en Guinée. Nous avons utilisé un outil de dépistage sérologique récemment mis au point, basé sur la technologie Luminex^®^, qui détecte simultanément plusieurs cibles. Les protéines ciblées étaient les nucléoprotéines (NP), les glycoprotéines (GP) (souches Mayinga et Kissidougou-Makona) et la protéine virale 40 (VP40).

## Résultats

Sur les 9 000 personnes prélevées dans l'EDS 2012, nous avons analysé les échantillons de 1 483 personnes parmi lesquelles 838 (56,5 %) provenaient des zones rurales de la Guinée, tandis que 645 (43,5 %) provenaient des villes. Après la sérologie, 154/1483 échantillons (10,4 %) ont réagi avec au moins un antigène EBOV. Cette proportion était de 4,1 % à Conakry, 19,4 % en Guinée forestière. Pris individuellement par antigène, 9,3 % des échantillons présentaient des anticorps IgG dirigés contre les GP d'EBOV et 9,3 % contre les protéines VP40 en Guinée forestière. Les échantillons collectés dans d'autres régions de Guinée présentaient également des anticorps IgG dirigés contre les protéines GP et VP40 d'EBOV, et la proportion variait entre 1 % et 5 %. Neuf échantillons (0,6 %) ont réagi contre les NP d'EBOV.

## Conclusion

Les résultats de cette étude montrent la présence d'anticorps anti-EBOV chez des personnes en Guinée 2 ans avant la survenue de l’épidémie de 2014-2016.

# Research article: Différence de séroprévalence mais séroneutralisation identique des anticorps anti-SARS-CoV-2 dans une population d’étudiants en médecine avec trois niveaux d'exposition distincts aux activités hospitalières à Bamako au Mali

**Published:** 2023-06-29

**Authors:** Yacouba CISSOKO, Issa KONATÉ, Djibril Mamadou COULIBALY, Daouda NAGNANGO, Djénéba FOFANA, Mama Adama TRAORÉ, Drissa KONÉ, Mariam SOUMARÉ, Dramane SOGOBA, Oumar MAGASSOUBA, Jean-Paul DEMBELÉ, Assétou FOFANA, Aminata MAIGA, Sounkalo DAO

**Affiliations:** 1 Service des maladies infectieuses et tropicales, CHU du Point G, Bamako, Mali; 2 Faculté de médecine et d'odontostomatologie (FMOS), Université des sciences, des techniques et des technologies de Bamako (USTTB), Bamako, Mali; 3 Laboratoire de biologie médicale, CHU du Point G, Bamako, Mali

## Introduction

La pandémie de Covid-19 débutée en décembre 2019 en Chine s'est rapidement propagée dans le monde, touchant le Mali fin mars 2020. La vaccination contre la Covid-19 étant d'actualité, nous avons voulu déterminer la séroprévalence anti-SARS-CoV-2 en milieu estudiantin médical avec différents degrés d'exposition.

## Méthodes

Il s'agit d'une étude castémoins qui s'est déroulée en mars 2022, comparant la séroprévalence des anticorps (IgA, IgM, IgG) anti-SARS-CoV-2 entre les étudiants non vaccinés en médecine et en odontostomatologie au Mali, répartis en trois groupes : étudiants en première année (sans activité hospitalière), externes (avec activité hospitalière intermittente) et internes (avec activité hospitalière continue).

## Résultats

L’âge moyen des étudiants était de 23,9 ± 3,7 ans. Le sexe masculin était majoritaire (80 %). Les gestes barrières étaient souvent utilisés par 93-98 % des étudiants. Dans les 3 groupes – étudiants en première année, externes et internes des hôpitaux – la séroprévalence des anticorps anti-SARS-CoV-2 de type IgA était respectivement de 11 %, 13 % et 24 %, celle des anticorps de type IgM de 11 %, 14 % et 23 % et celle des anticorps de type IgG de 12 %, 16 % et 24 %. Les internes avaient deux fois et demie plus de chance d'avoir une sérologie anti-SARS-CoV-2 positive que les étudiants en première année (OR = 2,4; IC [1,15-5,19]; p = 0,02). Cependant ces mêmes internes n'avaient une sérologie significativement plus positive que les externes seulement pour les IgA (OR = 2,11; [1-4,43]; p = 0,04). La majorité des étudiants séropositifs (97,7 %) possédait des anticorps anti-SARS-CoV-2 neutralisants.

## Conclusion

La séroprévalence du SARS-CoV-2 relativement faible chez les étudiants en médecine croît avec l'exposition à l'hôpital, d'où l'intérêt du renforcement de la vaccination.

# Research article: Place de la dengue parmi les états fébriles chez les patients consultant en milieu communautaire à Bamako

**Published:** 2023-06-29

**Authors:** Issa KONATÉ, Yacouba CISSOKO, I. GUINDO, H. MELI, Mariam SOUMARÉ, Dramane SOGOBA, Oumar MAGASSOUBA, Assètou FOFANA, F. BOUGOUDOGO, Soukalo DAO

**Affiliations:** 1 Service des maladies infectieuses et tropicales, CHU du Point G, Bamako, Mali; 2 Faculté de médecine et d'odontostomatologie (FMOS), Université des sciences, des techniques et des technologies de Bamako (USTTB), Bamako, Mali; 3 Faculté de pharmacie (FAPH), Université des sciences, des techniques et des technologies de Bamako (USTTB), Bamako, Mali; 4 Laboratoire de bactério-virologie, Institut national de santé publique, Bamako, Mali; 5 Centre universitaire de recherche clinique, FMOS/USTTB, Bamako, Mali

## Introduction

La dengue est une arbovirose émergente due à un virus appartenant à la famille des Flaviviridae. Sa présentation clinique est non spécifique, posant le diagnostic différentiel avec d'autres fièvres algiques notamment le paludisme.

## Objectif

Déterminer la place de la dengue chez les patients fébriles vus en consultation en milieu communautaire à Bamako.

## Méthodes

Il s'agissait d'une étude transversale chez les patients fébriles dans 4 centres de santé communautaires de Bamako en juillet 2021. Le diagnostic de dengue était basé sur la PCR et la sérologie. Les données ont été saisies et analysées grâce au logiciel SPSS 21.0. Les tests de Fisher et de Mann-Whitney ont été utilisés.

## Résultats

Sur 150 patients enrôlés, 62,7 % étaient de sexe féminin et la tranche d’âge de 11 à 20 ans représentait 28,7 % des cas. Les patients résidaient en commune 6 dans 60 % des cas. Outre la fièvre, les céphalées et les courbatures étaient retrouvées respectivement dans 82 et 71,3 %. L'AgNS1 était positif dans 1,3 % et les IgM et IgG respectivement dans 5,3 % et 8,3 % des cas. La dengue a été confirmée par la RT-PCR avec une positivité de 6 %, le taux de coinfection dengue-paludisme était de 55 %. Les facteurs environnementaux et le non-respect des mesures de lutte antivectorielle constituaient les déterminants majeurs de survenue de la dengue.

## Conclusion

Le virus de la dengue circule à Bamako, notamment en communes 5 et 6. Aussi convient-il de penser à ce diagnostic en cas de fièvre et de renforcer les mesures de prévention.

# Research article: Réintégration socioprofessionnelle des survivants d’Ébola dans les Districts sanitaires de Conakry, Coyah et Kindia

**Published:** 2023-06-29

**Authors:** >Gobou Tokpa NYANBALAMOU, Alexandre DELAMOU, Sâa Dimio SANDOUNO, Sidikiba SIDIBÉ, Bienvenue Salim CAMARA, B. S. Deltelphin KOLIE

**Affiliations:** 1 Service des maladies infectieuses et tropicales; 2 Chaire de santé publique, Hôpital national Donka, CHU de Conakry, Guinée; 3 Université Gamal Abdel Nasser de Conakry, Guinée

## Introduction

L'objectif de cette étude était d’évaluer la réintégration socioprofessionnelle des survivants d’Ébola dans les Districts sanitaires de Conakry, Coyah et Kindia.

## Matériel et méthodes

Il s'agissait d'une étude transversale de type descriptif s’étendant sur six (6) mois allant de mars à septembre 2015.

## Résultats

Au total, 131 survivants de la maladie à virus Ébola (MVE) ont été inclus dans l’étude. Ils avaient un âge médian de 35 ans et étaient majoritairement de sexe masculin (56,5 %o). Les étudiants et les professionnels de santé étaient les plus représentés avec 28,2 % et 25,2 % respectivement. 105 (80,2 %) vivaient avec leur famille ou partenaires avant la MVE, et ils étaient finalement 102 (77,9 %) après la maladie.

Le nombre des survivants qui vivaient seuls avant la MVE (5,3 %>) a augmenté depuis leur sortie du centre de traitement à (11,4 %). Le temps médian entre la sortie des survivants de la MVE du centre de traitement et leur interview était de 4,7 mois.

Plus de la moitié des survivants de la MVE ont affirmé être rejetés par des amis (66 %) et la communauté/le voisinage (55 %>). La majorité a affirmé être moins intégrée avec les amis (72 %), et/ou au lieu de travail/école (72 %) qu'avant la MVE. Ils ont affirmé que leur statut économique (90 %), leur situation de travail (80 %), et leur situation psychologique (60 %), étaient plus affectés depuis leur sortie du centre de traitement comparativement à la période avant la MVE.

Au total, 59 soit 45,5 % des survivants de la MVE étaient impliqués au moins dans une activité de lutte contre la MVE.

## Conclusion

Il y avait une nécessité de renforcer le suivi rapproché et prolongé des survivants de la MVE au sein de la communauté et dans les lieux de travail/école, afin de faciliter leur réintégration socioprofessionnelle.

# Research article: Étude des anomalies de l'hémostase chez les patients atteints de Covid-19 hospitalisés au Service de réanimation du Centre de traitement des épidémies (CT-ÉPI) de Donka

**Published:** 2023-06-29

**Authors:** Amadou Sékou CAMARA, Mamady DIAKITÉ, Mamadou Saliou SOW

**Affiliations:** 1 Service d'hématologie, Hôpital national Ignace Deen, Conakry, Guinée; 2 Service des maladies infectieuses, Hôpital national Donka, CHU de Conakry, Guinée

## Introduction

La Covid-19 peut s'accompagner de troubles de l'hémostase et est associée à une majoration du risque thrombotique. Cette étude avait pour objectif de contribuer à l'amélioration de la prise en charge des anomalies de l'hémostase au cours de la Covid-19 en Guinée par la description des types retrouvés.

## Matériel et méthodes

Il s'agissait d'une étude rétrospective de type descriptif et analytique d'une durée de 6 mois allant du 1^er^ août 2020 au 31 janvier 2021, réalisée au Service de réanimation du CT-ÉPI Donka.

## Résultats

Nous avons colligé 72 patients dont 60 (83 %) présentaient des anomalies de l'hémostase. L’âge moyen était de 60,5 ± 12 ans avec un sex-ratio de 2. Parmi nos enquêtés 48 (66,67 %) présentaient une augmentation des D-dimères et 12 (16,67 %) une thrombo-pénie. Sur 33 patients ayant réalisé le TP/INR, 18 (55,54 %) avaient présenté un TP abaissé avec augmentation de l'INR. À l’évaluation du risque d’évènement thromboembolique d'après la proposition du GFHT et GIHP, 29 (40,28 %) avaient un risque très élevé et 27 (37,50 %) un risque élevé. Au cours de l'hospitalisation, 4 de nos enquêtés ont présenté des complications hémorragiques. Nous avons trouvé un lien statistiquement significatif entre l'augmentation des D-dimères et la diminution des plaquettes par rapport à l’évolution avec des p-values à 0,0032 et 0,0002.

## Conclusion

Les anomalies de l'hémostase sont fréquentes chez les patients gravement atteints de Covid-19. La compréhension de ces anomalies pourrait permettre de leur apporter une prise en charge optimale.

# Research article: Paralysie faciale périphérique post-vaccinale à Covid-19 : à propos de deux cas observés au CHU de Conakry

**Published:** 2023-06-29

**Authors:** Mohamed Lamine CONDÉ, Souleymane Djigué BARRY, Mohamed Lamine TOURÉ, Kaba Malé CONDÉ, Idrissa Aissatou CAMARA, Mouloukou Souleymane DOUMBOUYA, Namory CAMARA, Fodé Abass CISSÉ

**Affiliations:** Service de neurologie, Hôpital national Ignace Deen, Conakry, Guinée

## Introduction

La paralysie faciale périphérique (PFP) est un symptôme fréquent, dominé par les formes idiopathiques. Celles d'origine toxique sont rares et peu documentées dans la littérature.

## Observation

Nous rapportons le cas de deux patients atteints d'une PFP de survenue brutale et rapidement résolutive dans les suites d'une vaccination à Covid-19. L'examen clinique et la recherche étiologique n'ont montré aucune cause spécifique. Le profil évolutif de cette paralysie faciale était compatible avec une cause toxique.

## Conclusion

Les paralysies faciales périphériques, bien que souvent idiopathiques, peuvent résulter d'une cause toxique. Le lien de causalité direct est souvent difficile à mettre en évidence et repose le plus souvent sur une démarche graduée basée essentiellement sur l'anamnèse et l'imagerie.

# Research article: Covid-19 au Mali : aspects épidémio-cliniques et évolutifs dans deux centres spécialisés de prise en charge en milieu urbain, Bamako, 2020-2021

**Published:** 2023-06-29

**Authors:** Abdoulaye Mamadou TRAORÉ, Modibo KEITA, Mamadou Karim TOURÉ, Bacary DIARRA, Salif SANAFO, Mamoudou KODJO, Yaya I. COULIBALY, Ousmane FAYE, Daouda K. MINTA

**Affiliations:** Service des maladies infectieuses et tropicales, CHU du Point G, Bamako, Mali

## Objectif

Décrire les caractéristiques cliniques, évolutives et les facteurs associés au décès au cours d'infection de Covid-19 à Bamako.

## Méthodologie

Il s'agit d'une étude transversale descriptive et analytique des patients Covid-19 pris en charge à l'Hôpital de dermatologie de Bamako et à la Clinique Pasteur de Bamako entre le 25 mars 2020 et le 31 décembre 2021. Le diagnostic a été confirmé principalement par RT-PCR.

## Résultats

Nous avons colligé 1351 dossiers de patients Covid-19, avec un âge moyen de 44,3 ans ± 17,9 et majoritairement de sexe masculin 56,44 % (786/1350). Les cas asymp-tomatiques représentaient 38,50 % (519/1348). Parmi les patients symptomatiques, les signes étaient modérés dans 46,14 % (522/1348) des cas; graves 13,65 % (184/1348); en revanche les patients avec des signes critiques représentaient seulement 1,71 % (23/1348). Les signes les plus fréquents étaient : toux (51,16 %), dyspnée (24,47 %), céphalées (19,67 %), anosmie (14,92 %), anorexie (11,77 %), écoulement nasal et agueusie (10,87 %). L'HTA (20,11 %) et le diabète (10,10 %) étaient les comorbidités prédominantes. Cinquante patients sont décédés (3,70 %). Les facteurs associés au décès sont : la gravité du tableau clinique, l’âge ≥ 60 ans (80 % vs 18 % pour âge < 60 ans, p < 0,001), l'existence de comorbidité de HTA (54 % vs 46 %, OR = 5,06, IC95% [2,84-9,07]) et le diabète (OR = 6,78, IC95% [3,68-12,32]).

## Conclusion

La symptomatologie de la Covid-19 est variable. Les personnes âgées de 60 ans et plus et avec comorbidité sont à risque de présenter les formes graves et critiques dont l'issue est le plus souvent fatale.

# Research article: Profil épidémioclinique du personnel de santé atteint de la Covid-19 au CT-ÉPI de Donka en 2020

**Published:** 2023-06-29

**Authors:** Souleymane BARRY, Mamadou Saliou SOW, Alpha KONÉ

**Affiliations:** 1 Service de cardiologie, CHU de Conakry, Guinée; 2 Service des maladies infectieuses et tropicales, CHU de Conakry, Guinée

## Introduction

La Covid-19 demeure l'une des maladies infectieuses dont les répercussions sociales et économiques sont particulièrement importantes. Cette étude avait pour objectif de décrire le profil épidémioclinique du personnel de santé atteint de Covid-19 au CT-ÉPI de Donka.

## Matériel et méthodes

Il s'agissait d'une étude rétrospective descriptive d'une durée de 6 mois (31 mars-1^er^ octobre 2020) réalisée au CT-ÉPI de Donka. Nous avons inclus dans cette étude tout le personnel de santé ayant une PCR positive à la Covid-19 hospitalisé au CT-ÉPI de Donka durant la période d’étude.

## Résultats

La fréquence de la Covid-19 chez le personnel de santé était de 5,8 %. Le sexe masculin était plus représenté soit 60,4 %, la profession la plus touchée était celle des médecins 43,2 % suivis des infirmiers 17,6 % et des biologistes 12,6 %, l’âge moyen des patients était de 43,79 ± 14,28 ans. Parmi nos patients, 39,1 % avaient été en contact avec une personne malade, 30,3 % travaillaient dans une structure sanitaire et 3,6 % travaillaient dans un CT-ÉPI. Les motifs de consultation les plus fréquents étaient l'asthénie 32,8 %, la céphalée 28,8 %, la fièvre 24,7 %, la toux 24,3 % et la dyspnée 16,2 %.

## Conclusion

Il ressort de cette étude que la prévalence de la Covid-19 chez le personnel de santé était faible. Le profil épidémiologique de la Covid-19 était représenté par les médecins, les infirmiers et les biologistes avec une prédominance masculine. La symptomatologie était polymorphe et la plupart des patients sont sortis guéris. Une étude similaire à l’échelle nationale pourrait aider à étudier davantage le profil épidémioclinique des personnels de santé atteints de la Covid-19 en Guinée.

# Research article: Évolution des variants du SARS-CoV-2 en Guinée de 2020 à 2022 : screening des mutations de la protéine Spike

**Published:** 2023-06-29

**Authors:** Haby DIALLO, Thibaut Armel Chérif GNIMADI, Aminata MBAYE, Abdoul Karim SOUMAH, Kadio Jean-Jacques Olivier KADIO, Cécé KPAMOU, Abdoulaye TOURÉ, Alpha Kabinet KEITA

**Affiliations:** 1 Centre de recherche et de formation en infectiologie de Guinée (CERFIG), Conakry, Guinée; 2 Institut de recherche pour le développement, IRD/UMI233/INSERM-U1175, Université de Montpellier, Montpellier, France

## Contexte

La pandémie de Covid-19 a rapidement évolué et a atteint des taux de contamination record dans le monde ainsi qu'en Guinée. Cette étude avait pour objectif de décrire l’évolution du SARS-CoV-2 par la détermination des variants circulant en Guinée.

## Matériel et méthodes

Les échantillons positifs de SARS-CoV-2 ayant un Ct (seuil de cycle) inférieur ou égal à 30 ont été analysés par la technique de criblage dans le cadre du projet AFROSCREEN. Cette méthode est basée sur la High Resolution Melting (fusion à haute résolution). Les échantillons ont été collectés chez les voyageurs et des patients suspects reçus dans les centres de traitement.

## Résultats

Au total, 301 échantillons prélevés entre novembre 2020 et août 2022 ont été analysés. Les résultats montraient que la transmission locale initiale était dominée par la souche d'origine (Wuhan) mais la deuxième vague a été largement dominée par le variant Alpha (B.1.1.7). Par la suite, le variant Delta (B.1.617.2) a circulé de mai à novembre 2021 avant d’être supplanté par le variant Omicron et ses sous-lignées, qui ont été introduits dans la transmission communautaire en décembre 2021. L'analyse mutationnelle a indiqué que les variants retrouvés avaient des substitutions au niveau de la Spike, telle que la mutation E484K, favorisant la transmission et l’échappement immunitaire.

## Conclusion

Cette étude a permis d'observer le potentiel d’émergence des variants du SARS-CoV-2 pendant les différentes vagues qui ont émergé en Guinée ainsi que dans d'autres régions du monde.

# Research article: Surveillance génomique des populations de SARS-CoV-2 isolées en Guinée : mutations et analyses phylogénétiques

**Published:** 2023-06-29

**Authors:** Thibaut Armel Chérif GNIMADI, Aminata MBAYE, Haby DIALLO, Kadio Jean-Jacques Olivier KADIO, Abdoul Karim SOUMAH, Cécé KPAMOU, Abdoulaye TOURÉ, Alpha Kabinet KEITA

**Affiliations:** 1 Centre de recherche et de formation en infectiologie de Guinée (CERFIG), Conakry, Guinée; 2 Institut de recherche pour le développement, IRD/UMI233/INSERM-U1175, Université de Montpellier, Montpellier, France

## Contexte

La pandémie de la Covid-19 reste encore une menace pour la santé à l’échelle mondiale. Le séquençage du génome et l'analyse bio-informatique du SARS-CoV-2 sont devenus des outils utiles dans la lutte contre la Covid-19. Nous rapportons les caractéristiques génétiques, les variations et l'analyse phylogénétique du SARS-CoV-2 en Guinée.

## Matériel et méthodes

Les échantillons étaient collectés chez des voyageurs et des patients suspects reçus dans les centres de traitement. Les kits DNAprep et COVIDSeq ont été utilisés pour la préparation des librairies. Le séquençage a été effectué à l'aide de la plateforme Illumina Iseq100 du laboratoire de génomique du Centre de recherche et de formation en infectiologie de Guinée (CERFIG) dans le cadre du projet AFROSCREEN. Les séquences sélectionnées avaient une couverture > 80 %.

## Résultats

Nos données portent sur 149 génomes et ont révélé 18 lignées différentes. Tous les génomes ont accumulé des mutations par rapport au Wuhan-Hu-1 (GenBank Accession No : MN908947.3). Selon l'ordre d'apparition des variants, nous avons détecté : Alpha (24), A.21 (01) et A.27 (01), B.1.629 (13), Eta (09), B.1.1.318 (08), R.1 (08), B.1 (06), AY.122 (02), Delta (02), Omicron et sous-lignées (75). Un total de 9602 sites de mutation nucléotidique a été trouvé dans 149 génomes. L'arbre phylogénétique des séquences obtenues et autres séquences du SARS-CoV-2 disponibles sur la plateforme internationale GISAID a été construit.

## Conclusion

Ces résultats fournissent une base scientifique pour retracer la source des infections et l’évolution du SARS-CoV-2 en Guinée et au-delà envisager une surveillance des contacts à partir de la génomique.

# Research article: Fréquence et facteurs associés à l'automédication contre la Covid-19 chez le personnel de santé de la ville de Conakry

**Published:** 2023-06-29

**Authors:** Abdoulaye TOURÉ, Kadio Jean-Jacques Olivier KADIO, Saidouba Cherif CAMARA, Alioune CAMARA, Alexandre DELAMOU, Salifou Talassone BANGOURA, Jean-François ÉTARD, Alpha Kabinet KEITA

**Affiliations:** 1 Centre de recherche et de formation en infectiologie de Guinée (CERFIG), Université Gamal Abdel Nasser de Conakry, Conakry, Guinée; 2 Chaire de santé publique, Université Gamal Abdel Nasser de Conakry, Conakry, Guinée; 3 Programme national de lutte contre le paludisme, Ministère de la Santé, Conakry, Guinée; 4 Centre d'excellence africain de prévention et de contrôle des maladies transmissibles, Université Gamal Abdel Nasser de Conakry, Conakry, Guinée; 5 TransVIHMI, Institut de recherche pour le développement, Université de Montpellier, INSERM, Montpellier, France

## Contexte

Les données concernant la prévalence et les conséquences de l'automédication pendant la pandémie de Covid-19 en Afrique sont très limitées. Cette étude avait pour objectif d'analyser la fréquence et les facteurs de risque de l'automédication contre la Covid-19 par le personnel de santé.

## Méthodes

Cette étude transversale s'est déroulée en juin 2021 dans les trois hôpitaux nationaux et les six centres médicaux communautaires, ainsi que dans cinq centres de santé primaires de la ville de Conakry. Un modèle de régression logistique multivarié a été réalisé pour identifier les facteurs associés à l'automédication.

## Résultats

Un total de 975 agents de santé d'un âge médian de 31 ans (IQR : 27-40), dont 504 (51,7 %) femmes ont été inclus. La majorité étaient des médecins (33,1 %) ou infirmières (33,1 %). Parmi toutes les personnes interrogées, 46,2 % ont déclaré avoir eu au moins un signe de Covid-19 au cours des 12 mois précédant l'enquête. La proportion d'automédication était de 15,3 % parmi le personnel des hôpitaux nationaux, de 12,20 % dans les centres médicaux des municipalités et de 22,6 % dans les centres de santé primaires (p = 0,06). Plus des deux tiers (68,7 %) des personnes qui se sont automédiquées n'ont pas subi de test de dépistage de l'infection par le SARS-CoV-2. Elles ont pris des antibiotiques, dont l'azithromycine, l'amoxicilline et l'ampicilline (42,2 %), ainsi que de l'acétaminophène (37,4 %), de la vitamine C (27,9 %), de l'hydroxychloroquine (23,8 %) et des plantes médicinales (13,6 %). La durée médiane de l'automédication était de 4 jours. La fatigue ou l'asthénie, le mal de gorge, la perte d'odorat et le mal de gorge d'une personne proche étaient indépendamment associés à l'automédication.

## Conclusion

Les professionnels de santé ont largement pratiqué l'automédication pendant la pandémie de Covid et sans test diagnostique. Les résultats suggèrent la nécessité de sensibiliser le personnel médical pour éviter les conséquences des molécules utilisées, notamment l'hépatotoxicité et la résistance aux antibiotiques.

# Research article: Étude de séroprévalence du SARS-CoV-2 chez le personnel soignant des hôpitaux de référence de Bamako : une étude prospective transversale multisites (ANRS COV11)

**Published:** 2023-06-29

**Authors:** Abdoulaye Mamadou TRAORÉ, Almoustapha Issiaka MAIGA, Amadou KODIO, Mahamadou SALIOU, Garan DABO, Philippe FLANDRE, Djénéba Bocar FOFANA, Karim AMMOUR, Nanzie Ornella Marie Emmanuelle TRA, Oumar DOLO, Anne-Geneviève MARCELIN, Ève TODESCO

**Affiliations:** Service des maladies infectieuses et tropicales, CHU du Point G, Bamako, Mali

## Introduction

Le but de cette étude était de déterminer la séroprévalence du SARS-CoV-2 chez les soignants dans les hôpitaux de référence de Bamako pendant la deuxième vague de Covid-19 (survenue début décembre 2020) en utilisant un TDR d'anticorps IgG et IgM, et de déterminer les facteurs prédictifs associés à une sérologie positive.

## Méthodes

Nous avons mené une étude de cohorte prospective, incluant les travailleurs de santé, âgés de 18 ans ou plus, présentant ou non des symptômes de Covid-19, exerçant dans les hôpitaux de référence de Covid-19 de Bamako (CHU Gabriel Touré, Hôpital de dermatologie de Bamako et Hôpital du Mali) ayant consenti à participer librement à l’étude. Le dispositif de test rapide Panbio™ IgG/IgM Covid-19 (Abbott Diagnostics) a été utilisé et couplé au test RT-PCR.

## Résultats

Un total de 200 participants dont 195 ont été sélectionnés : staff médical 37 %, paramédicaux 26,5 % et personnel administratif 36,5 %. L’âge médian est de 29 ans avec 57 % de sexe masculin. Une séroprévalence très élevée du SARS-CoV-2 de 51,8 % a été observée. Seuls 2 des 195 échantillons étaient positifs pour les IgM anti-nucléocapsides (1,0 %). Parmi les 2 échantillons, l'un était positif en IgG et l'autre négatif en IgG.

Les symptômes signalés au moment du dépistage étaient les suivants : rhinite (3,5 %), céphalées (3,0 %), toux (2,5 %), fièvre (1,5 %) et douleurs thoraciques (1,5 %). Vingt et un travailleurs de la santé ont déclaré avoir été en contact avec un cas confirmé de Covid-19 (10,5 %) et 29,2 % avaient été vaccinés contre la Covid-19 avec Vaxzevria^®^ (AstraZeneca) au moment du dépistage. Un nombre de personnes vivant dans le foyer (≥ 8 *versus* < 8) était un facteur prédictif indépendant d'une sérologie positive.

## Conclusion

Ces données confirment une large diffusion du virus SARS-CoV-2 dans la région. Dans ce contexte, le dépistage et la surveillance moléculaire doivent être renforcés et les professionnels de santé doivent bénéficier d'urgence d'une vaccination tant pour leur propre protection que pour la protection collective de la santé publique.

# Research article: Impact de la Covid-19 sur les activités de l'Hôpital national Donka (CHU de Conakry) et la prise en charge des PvVIH

**Published:** 2023-06-29

**Authors:** Ibrahima BAH, Mariama Boubacar DIALLO, Baba KALLÉ, Sidikiba SIDIBÉ, Mamadou Saliou SOW

**Affiliations:** 1 Université Gamal Abdel Nasser de Conakry, Conakry, Guinée; 2 Service des maladies infectieuses et tropicales, Hôpital national Donka, Conakry, Guinée

## Introduction

Cette étude avait pour objectif d’évaluer l'impact de la Covid-19 sur l'activité des urgences et des services médicaux de l'Hôpital national Donka.

## Méthodes

Il s'agissait d'une étude rétrospective de type descriptif d'une durée de 6 mois, 2 années de suite. L’étude a couvert une période allant du 1^er^ mars au 31 août 2019 (période avant Covid-19) et du 1^er^ mars au 31 août 2020 (période pendant Covid-19). Le test t de Student a été réalisé pour la comparaison des différences moyennes mensuelles.

## Résultats

Les données, résumées dans le tableau, ont montré une baisse de la consultation et de l'hospitalisation moyennes mensuelles en 2020 durant la pandémie de Covid-19 comparativement à 2019, avec une chute moyenne mensuelle de 275,17 passages au service des urgences (p = 0,005), 268,5 en dermatologie-MST (p = 0,012), 39,17 en maladies infectieuses et tropicales et 18,5 en médecine interne (p = 0,5). La différence moyenne mensuelle des hospitalisations est de 43,5 (p = 0,047); 4,83 (p = 0,011); 9,83 (p = 0,033) et 13,67 (p = 0,023) respectivement.

Nous avons aussi constaté une diminution significative (p = 0,001) de l'utilisation des services de santé pour les soins du VIH avec un total de 935 absents de mars à août 2020 contre 107 pour la même période en 2019 au Service des maladies infectieuses et tropicales, et 329 en 2019 contre 393 en 2020 au Service de dermatologie-MST.

Le déploiement du personnel pour la prise en charge de la pandémie a été plus marqué au Service des maladies infectieuses, suivi de celui de dermatologie-MST.

## Conclusion

La pandémie de Covid-19 a eu un impact négatif sur les services de santé, notamment la continuité des soins pour les PvVIH. Il serait nécessaire d'améliorer les stratégies de gestion de l'actuelle pandémie et le renforcement du système de santé notamment pour les pathologies chroniques.

# Research article: Impact psycho-social de la Covid-19 chez les guéris en milieu bancaire à Conakry

**Published:** 2023-06-29

**Authors:** Alhousseine YANSANÉ, Moussa KEITA, Sekou SOUARÉ, Marie Angèle N'DIAYE, Aminata DIALLO, Ansoumane MARA, Hassane BAH

**Affiliations:** 1 Faculté des sciences et techniques de la santé, Université Gamal Abdel Nasser de Conakry, Conakry, Guinée; 2 Service national de la médecine du travail, Km9 sur l'autoroute en face SOGEDI, Matam, Conakry, Guinée; 3 Service de médecine légale, CHU de Conakry, Guinée

## Introduction

La psychose de la Covid-19 était grandissante en milieu du travail avec des conséquences psycho-sociales importantes chez les travailleurs et sur le fonctionnement du service. L'objectif de l’étude était d’étudier l'impact psycho-social lié à la Covid-19 chez les guéris en milieu bancaire à Conakry.

## Matériel et méthodes

Il s'agissait d'une étude transversale de 6 mois, réalisée dans le secteur bancaire chez les salariés guéris de la Covid-19. Tous les salariés guéris de la Covid-19, disposant d'un certificat médical et ayant repris le travail ont été inclus. Les manifestations liées aux troubles du sommeil, au stress et à l'indice de capacité de travail ont été déterminées à l'aide des scores respectifs.

## Résultats

Sur un total de 1239 salariés, 112 guéris de la Covid-19 ont été contactés (9 %). Le sexe masculin représentait 72,3 %. L’âge moyen était de 43 ans et les mariés représentaient 68,7 %. La contamination entre collègues était retrouvée chez 81,3 % dont 8,9 % et 5,4 % souffraient respectivement de HTA et du diabète. Les manifestations psychosociales comprenaient le trouble modéré du sommeil chez 35,7 % et léger de l'anxiété chez 46,4 %, ainsi que le stress modéré chez 40,2 %. Certains (57 %) craignaient une réapparition des symptômes et d'autres (17 %) ont connu un accueil défavorable des collègues.

## Conclusion

La Covid-19 a eu un impact psycho-social sur la santé mentale des salariés des banques guéris. Il se traduisait par des manifestations psycho-sociales au cours de la maladie. La prise en charge multidisciplinaire des épidémies favorise la gestion des facteurs psycho-sociaux et professionnels des malades.

# Research article: Séroprévalence associée au portage de l'AgHBs en milieu carcéral guinéen

**Published:** 2023-06-29

**Authors:** Aboubacar DIAKITÉ, Dembo DIAKITÉ, Mamadou Oury Safiatou DIALLO, Ibrahima BAH, Saidouba Cherif CAMARA, Ibrahima CAMARA, Édouard Florent BANGOURA, Moussa BALDÉ, Lansana CAMARA, Ansoumane CAMARA, Fodé Bangaly SAKO, Fodé Amara TRAORÉ, Mamadou Saliou SOW

**Affiliations:** 1 Service des maladies infectieuses et tropicales, Hôpital national Donka, Conakry, Guinée; 2 Centre de recherche et de formation en infectiologie de Guinée (CERFIG), Conakry, Guinée

## Objectif

Déterminer la prévalence et les facteurs associés au portage de l'AgHBs en milieu carcéral guinéen.

## Matériel et méthodes

Étude observationnelle transversale, multicentrique descriptive et analytique qui s'est déroulée sur une période de 6 mois dans les prisons centrales des différentes régions administratives de la Guinée. Nous avons inclus les détenus de tout âge, de tout sexe ayant accepté de participer à l’étude et chez lesquels le dépistage de l'hépatite virale B a été réalisé. Les informations socio-démographiques, cliniques et biologiques ont été collectées. L'AgHBs a été recherché sur les prélèvements de sang veineux grâce au test rapide CYPRESS AgHBs BANDELETTES^®^. Les facteurs associés au portage de l'AgHBs ont été analysés à l'aide d'une régression logistique multivariée.

## Résultats

Sur 873 détenus, 153 soit une prévalence de 17,5 %, IC95% [15,1-19,8] étaient porteurs de l'AgHBs. L’âge moyen a été de 29,70 ± 10,08 ans, le sexe masculin 96,9 %. Les proportions de l'AgHBs étaient respectivement 21,7 et 20,5 % à Boké et Kindia. L'analyse logistique multivariée a révélé que la durée de détention de 5-10 ans (OR = 2,20, IC95% [1,05-4,63], p = 0,03), de plus de 10 ans (OR = 4,08, IC95% [1,49-11,18], p < 0,01), le partage de lame entre détenus (OR = 4,08, IC95% [2,07-4,38], p < 0,01), l'usage de cocaïne (OR= 7,75, IC95% [1,66-36,09], p < 0,01) étaient indépendamment associés au portage de l'AgHBs.

## Conclusion

La prévalence de l'infection à VHB parmi les détenus reste élevée. Les facteurs indépendamment associés au portage de l'AgHBs ont été les détenus de plus de 5 ans, le partage de lame et l'usage de cocaïne. La lutte contre cette affection en milieu pénitentiaire nécessite un dépistage, une sensibilisation et une réforme de l'offre des soins. Des études plus approfondies sur la circulation virale et l'impact du milieu carcéral sur l'infection par le VHB semblent nécessaires.

# Research article: Covid-19 au Centre d'hémodialyse de l'Hôpital national Donka

**Published:** 2023-06-29

**Authors:** Mamadou Saliou BALDE, Fousseny DIAKITÉ, Moussa TRAORÉ, Aly TRAORÉ, Mamadouba CAMARA, Mohamed Lamine KABA, Alpha Oumar BAH

**Affiliations:** Service de néphrologie-hémodialyse, Hôpital national Donka, Conakry, Guinée

## Introduction

Les patients souffrant d'une insuffisance rénale chronique terminale doivent continuer de recevoir leur séance d'hémodialyse malgré l'infection à Covid-19. Le déplacement d'un hémodialysé de chez lui au centre occasionne des croisements avec d'autres personnes qui pourraient être infectées par le SARS-CoV-2. En Guinée, l'Agence nationale de sécurité sanitaire (ANSS) a émis des recommandations sur les mesures barrières ainsi que sur la protection du personnel en service dans le milieu hospitalier. L'objectif de notre étude était de déterminer la prévalence de l'infection à Covid-19 dans le Centre national d'hémodialyse.

## Méthodes

Nous avons fait une étude obser-vationnelle d'une durée de 4 mois allant du 15 avril au 15 août 2020. Nous avons inclus tous les patients infectés et le personnel soignant déclaré positif à l'infection Covid-19 après obtention de leur accord. Les paramètres sociodémographiques, le déroulement des séances de dialyse, l'association à une comorbidité et la durée d'hospitalisation ont été évalués.

## Résultats

Sur les 240 personnes du Centre, nous avons suspecté 13 personnes dont 8 ont été positives à l'infection Covid-19 soit une fréquence de 3,92 %. Les personnes positives étaient composées de 3/190 (1 %) patients hé-modialysés et 5/50(10 %) personnels soignants. La durée de séjour moyenne en hospitalisation était de 7 ± 3 jours pour le personnel soignant et 15 ± 5 jours pour les patients hémodialysés.

## Conclusion

Le Centre national d'hémodialyse a connu un taux d'infection relativement faible. L'infection à Covid-19 est une pandémie mondiale à laquelle le Centre national d'hémodialyse devrait s'adapter à travers le strict respect des mesures barrières, tout en augmentant le nombre de tests afin de détecter les personnes à risques.

# Research article: La tuberculose pulmonaire à l’ère de la pandémie à Covid-19 : aspects épidémiologiques, cliniques et évolutifs

**Published:** 2023-06-29

**Authors:** Mamoudou SAVADOGO, Ismaël DIALLO, Kognimisson Apoline SONDO, Kader DAO, Djenaba LY, Arsène OUÉDRAOGO

**Affiliations:** 1 Service des maladies infectieuses, CHU Yalgado Ouédraogo, Ouagadougou, Burkina Faso; 2 Service de médecine interne, CHU Yalgado Ouédraogo, Ouagadougou, Burkina Faso

## Introduction

L'année 2019 a été marquée par l’émergence de la Covid-19. Cette étude avait pour objectif de décrire les caractéristiques épidémiologiques, cliniques et évolutives de la tuberculose pulmonaire à l’ère de la pandémie de Covid-19.

## Patients et méthodes

Il s'agit d'une étude transversale prospective à visée descriptive durant trois mois, du 1^er^ mars au 30 mai 2021. Ont été inclus les patients suspects de Covid-19 admis au Service des maladies infectieuses du CHU Yalgado Ouédraogo, chez qui la tuberculose pulmonaire a été diagnostiquée à partir des arguments cliniques, biologiques et/ou radiologiques.

## Résultats

Durant la période d’étude, 33 cas suspects de Covid-19 avaient été reçus dans le Service des maladies infectieuses du CHU Yalgado Ouédraogo. Parmi eux, 21 cas de tuberculose pulmonaire ont été diagnostiqués. L’âge moyen des patients tuberculeux était de 45 ans avec des extrêmes de 20 ans et 72 ans. Le sexe masculin dominait (13) contre 8 pour le sexe féminin, soit un sex-ratio de 1,63. Les patients provenaient majoritairement de la province du Kadiogo (71,4 %). Parmi les patients tuberculeux, 4 souffraient de tuberculose confirmée bactériologiquement dont 1 cas positif au SARS-CoV-2. La toux était présente chez 13 patients, une dyspnée retrouvée chez 10 patients, une douleur thoracique était présente chez 4 patients, une douleur abdominale, une anorexie et des vertiges retrouvés respectivement chez 2 patients. À l'examen physique, un syndrome infectieux était présent chez 10 patients, une altération de l’état général retrouvée chez 9 patients, un syndrome d’épanchement liquidien était retrouvé chez 8 patients, un syndrome de condensation pulmonaire était noté chez 5 patients et un syndrome d’épanchement gazeux chez 2 patients. Les comorbidités étaient dominées par l'infection à VIH (4 cas). L’évolution sous traitement a été marquée par le décès de 4 patients soit une létalité de 23,5 %.

## Conclusion

La pandémie de Covid-19 semble avoir induit une augmentation du nombre des cas de tuberculose pulmonaire. Il importe de penser aussi à la tuberculose chez tout patient suspect de Covid-19 eu égard à la similitude de certains signes respiratoires.

# Research article: Covid-19 chez les personnes vivant avec le VIH admises à l'Unité de soins, de formation et de recherche de l'Hôpital national Donka de Conakry

**Published:** 2023-06-29

**Authors:** Fodé Bangaly SAKO, Thierno Boubacar BARRY, Boubacar BALDÉ, Camara KELETY, Mohamed Marie DOUMBOUYA, Ibrahima BAH, Mamadou Oury Safiatou DIALLO, Fodé Amara TRAORÉ, Mamadou Saliou SOW, Mohamed CISSÉ

**Affiliations:** 1 Service des maladies infectieuses et tropicales, Hôpital national Donka, Conakry, Guinée; 2 Université Gamal Abdel Nasser de Conakry, Conakry, Guinée; 3 Université Kofi Annan de Guinée, Conakry, Guinée; 4 Service de dermatologie-MST/Unité de soins, de formation et de recherche, Hôpital national Donka, Conakry, Guinée

## Introduction

La pandémie de Covid-19 est rapidement devenue une menace importante pour la santé publique. Les personnes vivant avec le VIH sont confrontées à de multiples morbidités qui pourraient augmenter les conséquences graves liées à l'infection par le SARS-CoV-2.

L'objectif de cette étude était de déterminer le profil clinique, immuno-virologique et évolutif des personnes vivant avec le VIH atteintes de Covid-19.

## Matériel et méthodes

Il s'agissait d'une étude observationnelle et analytique portant sur les personnes vivant avec le VIH (PvVIH) admises dans l'Unité pendant une période de 11 mois (du 1^er^ mai 2021 au 31 mars 2022).

## Résultats

Sur un total de 727 PvVIH testées à l'admission, 102 étaient positives à la Covid-19, soit 14 %. La moyenne d’âge était de 39 ± 12,106 ans [20-75 ans] avec une prédominance féminine (61 %). La fièvre (73 %), la toux (67 %) et l'asthénie physique (41 %) étaient les symptômes les plus courants chez ces PvVIH. L'infection opportuniste la plus fréquente était la tuberculose (70 %) et la comorbidité la plus associée était l'insuffisance rénale (43 %). Il s'agissait d'une infection par le VIH de type 1 dans 99 % des cas avec un stade clinique III de l'OMS dans 28 % et IV dans 72 %. Dans 56 % des cas, le taux de CD4 était inférieur à 100 cell./mm^3^ avec une moyenne de 118,01 ± 140,36 cell./mm^3^ [8-995 cell./mm^3^] et 78 % avaient une charge virale ≥ 50 copies/ml. Nous avons enregistré 30 cas de décès soit 29 % dont les facteurs associés étaient la tuberculose pulmonaire [p = 0,057], la pneumocystose [p = 0,018], l'insuffisance rénale [p = 0,006], les hépatites [p = 0,075], la détresse respiratoire [p = 0,001] et le sepsis [p = 0,080].

## Conclusion

Il ressort de notre étude que la survenue de la Covid-19 est associée à de multiples affections émaillées de complications et de décès chez les PvVIH à un stade avancé de l'infection.

# Research article: Mortalité des patients Covid-19 avec comorbidités de la région de Labé

**Published:** 2023-06-29

**Authors:** Sadou SOW, Alpha Oumar DIALLO

**Affiliations:** Université Gamal Abdel Nasser de Conakry, Conakry, Guinée

## Contexte

Depuis 2019, le monde souffre de la pandémie de maladie à coronavirus (Covid-19). Nous avons entrepris une étude pour documenter les facteurs associés à la mortalité des patients Covid-19 ayant des comorbidités dans la région de Labé.

## Méthodes

Il s'agissait d'une étude transversale de type descriptif et analytique à partir des dossiers et de la base de données des patients hospitalisés pour Covid-19 dans la région de Labé. Pour la saisie et l'analyse des données, les logiciels du Pack Office 2016, le logiciel R et les tests statistiques chi2 (p < 0,05) ont été utilisés.

## Résultats

Sur 1443 patients de Covid-19 qui ont participé à notre étude, 244 avaient des comorbidités et 1199 patients de Covid-19 étaient indemnes des comorbidités. L’âge moyen des patients était de 45 ans avec des extrêmes de 7 à 99 ans et le sex-ratio était de 1,33 en faveur des hommes.

L'hypertension artérielle était la plus fréquente avec 56,55 % des patients, suivie du diabète avec 46,72 % des cas; 5,32 % avaient le VIH et les cancers concernaient 1,63 % des patients Covid-19 avec comorbidités. Selon le statut clinique, 56 % des patients avec comorbidités étaient des cas sévères. La durée d'hospitalisation était globalement de 21 jours. Le taux de mortalité était de 12 % chez les patients ayant des comorbidités et de 3 % chez les autres (p < 0,005). Parmi les patients ayant des comorbidités, 8,19 % sont décédés du diabète; 5,73 % de l'hypertension artérielle; 2 % de l'association diabète et hypertension artérielle; et 0,4 % du VIH.

## Conclusion

Les facteurs de mortalité liée au Covid-19 dans la région de Labé sont l’âge, la présence de comorbidités en particulier l'hypertension artérielle, le diabète, le VIH et les maladies pulmonaires chroniques. Ces facteurs prédictifs pourraient aider les cliniciens à identifier les patients ayant un mauvais pronostic à un stade précoce, afin de réduire la mortalité liée à la Covid-19. Par conséquent, il faudra prioriser les sujets âgés et tarés dans la mise en œuvre des mesures préventives. Une stratégie de vaccination efficace peut améliorer considérablement l'espérance de vie, remodelant ainsi les composantes de la société et de l’économie.

# Research article: Évolution de la file active des personnes vivant avec le VIH (PvVIH) suivies au Service des maladies infectieuses et tropicales (SMIT) du CHU de Treichville dans le contexte de la pandémie à Covid-19, Abidjan, Côte d'Ivoire

**Published:** 2023-06-29

**Authors:** Salif DIAWARA, Affoué Gisèle KOUAKOU, Melaine Chrysostome MOSSOU, Jérémie KINNOUDO, Sara N'DAW, Hermann Nguessan FAITEY, Marie Nicole KONAN, Nogbou Frédéric ELLO, Ndouba Alain KASSI, Adama DOUMBIA, Wadartou Dine MOURTADA, Bernice Corinne AKPOVO, Eboi EHUI, Koffi Aristophane TANON, Serge Paul EHOLIÉ

**Affiliations:** 1 Unité de formation et de recherche des sciences médicales, Abidjan, Côte d'Ivoire; 2 Service des maladies infectieuses et tropicales, Centre hospitalier universitaire de Treichville, Abidjan, Côte d'Ivoire

## Introduction

Virose émergente qualifiée par l'OMS comme une menace sanitaire internationale, la maladie à coronavirus constitue une menace pour le continuum des soins chez les PvVIH. Les obstacles et défis se posent pour le suivi des PvVIH dans ce contexte de crise sanitaire.

## Objectif

Apprécier l'effet de la pandémie à Covid-19 sur la file active des PvVIH suivies au SMIT.

## Matériel et méthodes

Étude transversale réalisée au SMIT de mars 2020 à mars 2021. Les données ont été recueillies à partir des rapports mensuels et de la base électronique du SMIT. Le profil évolutif de la file active, les caractéristiques sociodémographiques, les données biologiques et thérapeutiques ont été analysés.

## Résultats

La file active attendue à mars 2021 est passée de 4 533 patients à 4074. Sur la période d’étude, nous avons notifié 722 patients déclarés perdus de vue dont 283 ont été retrouvés, 13 décès et 7 transferts. La Population résidait majoritairement dans le grand Abidjan. Seulement 216 patients ont initié un suivi et 1236 charges virales réalisées pour les patients en cours de suivi avec 1002 patients en succès virologique. Le taux de régularité à la pharmacie antirétrovirale a baissé en dessous de 50 % après mars 2020.

## Conclusion

Des moyens innovants permettant d'assurer un suivi à distance et un bon accompagnement psycho-social doivent être déployés d'urgence pour un contrôle anticipé de ces perturbations liées à la pandémie à Covid-19 sur le moyen et long terme chez les PvVIH.

# Research article: Séroprévalence de l'infection par le SARS-CoV-2 à la prison centrale de Conakry, Guinée

**Published:** 2023-06-29

**Authors:** Mamadou Mouminy BARRY, Mamadou Saliou SOW, Frédéric LE MARCIS, Thibaut Armel Chérif GNIMADI, Yaya FOFANA, Kadio Jean-Jacques Olivier KADIO, Alpha Kabinet KEITA, Abdoulaye TOURÉ

**Affiliations:** 1 Centre de recherche et de formation en infectiologie de Guinée (CERFIG), Faculté des sciences et techniques de la santé, Université Gamal Abdel Nasser de Conakry, Conakry, Guinée; 2 Service des maladies infectieuses et tropicales, Hôpital national Donka, Conakry, Guinée

## Introduction

Les prisons en Afrique sont considérées comme des facteurs de risque de transmission des virus respiratoires en raison de la surpopulation et de la promiscuité. Le but de cette étude était d'estimer la séropré-valence de l'infection par le SARS-CoV-2 à la prison centrale de Conakry.

## Méthodes

Une étude descriptive a été menée sur une période d'un mois allant du 10 août au 10 septembre 2020 chez les détenus et le personnel de la prison centrale de Conakry. Les données collect**ées sur une fiche d'enquête** pré-**établie** ont été décrites sous forme de médiane avec interquartiles pour les variables quantitatives et sous forme de proportions pour les variables qualitatives. La tendance d'hétérogénéité a été vérifiée à l'aide de SPSS-21 et le reste de l'analyse a été effectué avec Epi Info-7.2.5.0. Les participants ont été diagnostiqués par un test de diagnostic rapide (GoldMag SARS-CoV-2 IgG/IgM Antibody Testing Kit) pour la recherche des anticorps IgM/IgG dirigés contre le SARS-CoV-2.

## Résultats

Au total, 156 participants ont bénéficié d'un test de diagnostic rapide au cours de l’étude. Sur l'ensemble des détenus, 70 % étaient des prévenus. L’âge médian était de 32 ans (25-37). Trois quarts de l’échantillon étaient des hommes et 60 % étaient célibataires. La durée médiane d'incarcération était de 9 mois avec des extrêmes de 1 et 169 mois. La majorité des détenus provenait des communes de Matoto et de Ratoma. Les participants ont affirmé avoir présenté des céphalées (25,0 %) et la gale (12,2 %). La séroprévalence de l'infection par le SARS-CoV-2 était de 74 % (IC95% = 66,6-80,8). Soixante-douze pour cent des hommes étaient séropositifs contre 28 % chez les femmes et l’âge médian des enquêtés séropositifs était de 32 ans.

## Conclusion

Cette étude a permis de savoir qu'une transmission rapide et généralisée du SARS-CoV-2 a eu lieu à prison centrale de Conakry. Une étude complémentaire généralisée sur l'ensemble de la population carcérale est nécessaire pour cerner la dynamique de séro-conversion du SARS-CoV-2 dans l'ensemble des établissements pénitentiaires du pays.

# Research article: Prévalence de la Covid longue et ses manifestations chez les patients traités à Parakou en 2020-2021

**Published:** 2023-06-29

**Authors:** Cossi Angelo ATTINSOUNON, Patrice MONYÈ, Léopold CODJO, Serge ADÉ, Julien ATTINON, Rokhiatou BABIO, Ibrahim Mama CISSÉ

**Affiliations:** 1 Site de prise en charge de la Covid-19 de l'Hôpital d'instruction des Armées de Parakou, Bénin; 2 Département de médecine et spécialités médicales, Faculté de médecine, Université de Parakou, Bénin

## Introduction

La Covid-19 devient une maladie chronique chez plusieurs patients. Ce travail avait pour objectif de calculer la prévalence de Covid longue et de décrire ses manifestations chez les patients suivis à Parakou en 2020-2021.

## Méthode

Une étude transversale s'est déroulée du 1^er^ avril au 30 juin 2022. Tous les patients hospitalisés du 8 mai 2020 au 31 décembre 2021 pour une infection au SARS-CoV-2 confirmée et sortis vivants ont été inclus. Un questionnaire a été administré aux enquêtés et certaines données ont été recueillies dans les dossiers médicaux. La variable d'intérêt était la présence de manifestations cliniques relatives à la Covid longue. Les données ont été saisies dans un masque sur KoboCollect v2022.1.2 puis analysées par Epi Info.

## Résultats

Au total, 113 patients ont été inclus dans cette étude sur les 163 patients sortis vivants du site. L’âge moyen était de 50,25 ± 19,30 ans et le sex-ratio de 1,40. La prévalence de la Covid longue était de 56,64 % (64 patients). Les principaux symptômes de la Covid longue étaient la fatigue (56; 87,5 %), l'essoufflement (29; 45 %), l'insomnie (15; 23,44 %), la toux (14; 21,88 %), l'anxiété (13; 20,31 %) et la dépression (13; 20,31 %). Après la sortie du site, 17 patients sont décédés (15,05 %). Les facteurs associés à la Covid longue étaient l’âge ≥ 50 ans (p = 0,037), la profession (p = 0,026), avoir plus de cinq symptômes à l'admission (p = 0,017), la gravité de la Covid-19 (p = 0,026), le séjour en réanimation (p = 0,008) et la durée d'hospitalisation ≥ 15 jours (p = 0,049).

## Conclusion

La prévalence de la Covid longue est élevée à Parakou. La mise en place d'un suivi des patients survivants de la Covid-19 s'impose pour faciliter leur retour à un état de santé normal.

# Research article: Abcès cérébral sur terrain de cardiopathie congénitale : Étude d'un cas avec revue de la littérature

**Published:** 2023-06-29

**Authors:** Yakhouba FOFANA, Mohamed CHERIF, Mohamed Lamine SYLLA, Oumar SOW, Élisabeth TRAORÉ, Fatoumata Binta KEITA, Morifodé SOUMAH, Mamadou Atigou DRAME, Fallilou CAMARA, Ibrahima Sory SOUARÉ

**Affiliations:** Service de neurochirurgie, Hôpital de l'amitié sino-guinéenne (HASIGUI), Kipé, Guinée

Les abcès cérébraux sont pourvoyeurs d'une importante morbidité chez les patients atteints de cardiopathie congénitale. Dans les pays à ressources limitées, leur prise en charge se heurte à de nombreuses difficultés et le pronostic est souvent réservé faute de prise en charge adéquate. Nous rapportons ici un cas d'abcès cérébral sur terrain de cardiopathie congénitale (canal atrio-ventriculaire) à l'Hôpital de l'amitié sino-guinéenne (République de Guinée). Il s'agit d'une fille de 11 ans, référée dans notre service pour des crises convulsives, une hémiparésie gauche. L'interrogatoire et l'examen physique avaient permis de retrouver une symptomatologie évoluant depuis deux (2) mois faite de céphalées persistantes, de fièvre, de difficultés respiratoires, de vomissements et d'une impotence fonctionnelle de l'hémicorps gauche. Un état général altéré et une hémiparésie gauche. Le scanner cérébral avait montré un processus tissulaire arrondi à paroi épaisse se rehaussant après injection du produit de contraste, le tout associé à un important œdème péri-lésionnel responsable d'un engagement sous-falcoriel, faisant évoquer un abcès en région pariétale droite. L’écho-doppler cardiaque avait confirmé l'existence d'un canal atrio-ventriculaire avec une fuite mitrale et tricuspidienne modérée à moyenne. L'indication chirurgicale d'une trépano-ponction a été posée après la prise en charge de sa décompensation respiratoire. Les abcès cérébraux constituent une complication rare des cardiopathies congénitales. Leur issue est fatale en l'absence de traitement adéquat, d'où l'intérêt d'un diagnostic et d'une prise en charge précoce de ces cardiopathies.

# Research article: Abcès cérébelleux d'origine otogène : À propos d'un cas

**Published:** 2023-06-29

**Authors:** Mamady CAMARA, Karinka DIAWARA, Louceny Fatoumata BARRY, Seny YOULA, Ibrahima Sory KONATÉ, Mohamed Salif CAMARA, Mamadi Sekou CONDÉ, Laila TOURÉ, Abdoul Bachir DJIBO HAMANI, Souleymane Djigué BARRY, Kaba CONDÉ, Fodé Abass CISSÉ

**Affiliations:** 1 Service de neurologie, Hôpital national Ignace Deen, CHU de Conakry, Guinée; 2 Service de rhumatologie, Hôpital national Ignace Deen, CHU de Conakry, Guinée

## Introduction

L'abcès cérébelleux d'origine otogène est la conséquence d'une infection auriculaire non ou insuffisamment traitée. Actuellement, on observe une diminution de l'incidence et une bonne évolution grâce au progrès de l'imagerie mais aussi de nouvelles générations d'antibiotiques. Cependant les complications sont rares mais graves. L'objectif était de rapporter un cas d'abcès cérébral d'origine otogène.

## Observation

Il s'agissait d'une patiente de 60 ans, ménagère aux antécédents d'otite moyenne chronique. Elle a présenté sur une période de 19 jours une otalgie, une tuméfaction, un écoulement purulent de l'oreille gauche et une fièvre. L'examen neurologique a révélé un syndrome cérébelleux et un syndrome d'hypertension intracrânienne.

Le scanner cérébral avec injection du produit de contraste a mis en évidence une image en cocarde aux dépens de l'hémisphère cérébelleux gauche et une hydrocéphalie triventri-culaire, faisant évoquer un abcès.

Nous avons observé une amélioration des signes après une antibiothérapie suivie du drainage des abcès. La culture du pus postopératoire était négative.

## Conclusion

L'abcès cérébelleux d'origine otogène est rare et grave. Le pronostic dépend de la précocité de la prise en charge.

# Research article: Acteurs et pratiques de la PCI dans les structures de santé

**Published:** 2023-06-29

**Authors:** Amadou Tidiane BARRY

**Affiliations:** Centre de recherche et de formation en infectiologie de Guinée (CERFIG), Conakry, Guinée

Depuis la survenue de la maladie à virus Ébola (MVE) en Guinée (2013-2016), le gouvernement et ses partenaires ont mis en œuvre plusieurs actions dans le cadre de la prévention et du contrôle des infections (PCI) dans les structures de santé. Cela se traduit par la fourniture du matériel et la formation du personnel. Comment ces actions se matérialisent-elles sur le terrain ? Cette question m'a poussé à m'intéresser à un certain nombre de pratiques à l'Hôpital national Ignace Deen et à l'Hôpital régional de Kankan.

Les informations discutées dans cette présentation sont issues d'ethnographies réalisées dans le cadre du programme PCI de long cours au sein de ces structures de santé. Les observations ont porté sur : le nettoyage, l'offre de soins, les interventions chirurgicales, les rondes, la supervision et les séances de formation. Quant aux entretiens, d'une part, ils ont porté sur le parcours professionnel de chaque acteur. D'autre part, les échanges ont porté sur la perception des acteurs concernant : les formations en PCI, la pratique de la PCI dans leurs structures, leurs propres pratiques et rôles dans la PCI.

Ces données révèlent que les plus exposés au risque d'infection sont dans une certaine mesure les moins impliqués dans les formations en PCI. En effet, on peut dire que les médecins et les infirmiers sont formés; les agents d'entretien sont orientés et les garde-malades sont quasiment ignorés dans le cadre de la prévention et du contrôle des infections. Par ailleurs, les formations ne suffisent pas pour une application véritable des notions en PCI. La pratique des règles dépend du parcours et des ambitions du responsable de la structure ou du service. Elle est aussi respectée de façon circonstancielle pour l'image de la structure. Enfin, on peut noter que l'insuffisance d'intrants empêche l'application de la PCI.

# Research article: Mésusage et risque de surdosage aux antibiotiques dans un contexte d'automédication en période Covid-19 à Conakry (MRSAA-Covid-19)

**Published:** 2023-06-29

**Authors:** Kadio Jean-Jacques Olivier KADIO, Djané Guillaume KADIO, Sidikiba SIDIBÉ, Adrien Fapeingou TOUNKARA, Abdoulaye TOURÉ

**Affiliations:** 1 Centre de recherche et de formation en infectiologie de Guinée (CERFIG), Conakry, Guinée; 2 Chaire de santé publique et législation pharmaceutique, Département des sciences pharmaceutiques et biologiques, Université Gamal Abdel Nasser de Conakry, Guinée; 3 Chaire de galénique et évaluation des médicaments, Département des sciences pharmaceutiques et biologiques, Université Gamal Abdel Nasser de Conakry, Guinée; 4 Chaire de santé publique, Département de médecine, Université Gamal Abdel Nasser de Conakry, Guinée; 5 Centre d'excellence africain de prévention et de contrôle des maladies transmissibles, Université Gamal Abdel Nasser de Conakry, Guinée; 6 Programme national de lutte contre le VIH/sida et les hépatites, Conakry, Guinée

## Introduction

La prise d'antibiotiques en automédication pourrait conduire à une résistance antimicrobienne, une intoxication médicamenteuse voire la mort. Le but de notre étude était d'analyser l'usage et le risque de surdosage des antibiotiques pris en automédication dans les officines privées de Conakry.

## Méthodes

Nous avons mené une étude transversale de juin à septembre 2021 dans des pharmacies d'officines privées des cinq communes de la ville de Conakry. Les clients adultes (≥ 18 ans) ayant acheté des antibiotiques en automédication ont été invités à participer à l'enquête. Les réponses ont été analysées individuellement pour chacune des molécules d'antibiotiques à travers trois (3) scores : usage, connaissance et risque de surdosage.

## Résultats

Au total, 73 officines privées ont été visitées et 1088 personnes venues en officine pour l'achat d'antibiotiques (amoxicilline, ciprofloxacine ou azithromycine) en vente libre ont été interrogées. Près de 64 % des participants faisaient recours à l'automédication au moins 4 à 5 fois par an et 27 % au moins une fois par mois. Concernant l'amoxicilline, 26,2 % étaient bons usagers, moins de 50 % avaient une bonne connaissance et environ 62 % étaient à risque probable de surdosage. S'agissant de la ciprofloxacine, moins d'un quart étaient bon usagers, 38 % avaient une bonne connaissance et 89 % étaient à risque de surdosage. Pour l'azithromycine, 39 % étaient bons usagers, 40 % avaient de bonnes connaissances et plus des trois quarts étaient à risque de surdosage.

## Conclusion

Notre étude a mis en évidence des proportions faibles de bons usagers, de bonnes connaissances et des proportions au-delà de 50 % en situation potentielle de risque de surdosage pour l'amoxicilline, la ciprofloxacine et l'azithromycine. Il paraît donc nécessaire de rappeler aux agents de santé le rôle important qu'ils ont à jouer dans l'utilisation des antibiotiques en automédication afin de réduire le risque de résistance aux antimicrobiens.

# Research article: Caractéristiques des patients et sensibilité des bactéries uro-pathogènes au Centre hospitalier Marguerite de Lorraine

**Published:** 2023-06-29

**Authors:** Karamba SYLLA, Mamadou Ciré BALDÉ, Loïc EPELBOIN, Félix DJOSSOU

**Affiliations:** 1 Service des maladies infectieuses et tropicales, CHU de Conakry, Guinée; 2 Unité des maladies infectieuses et tropicales, Centre hospitalier de Cayenne, Guyane, France; 3 Centre hospitalier Marguerite de Lorraine, Mortagne-au-Perche, France

## Introduction

Principales raisons d'explorations microbiologiques et d'utilisation intensive des antibiotiques, les infections urinaires demeurent un problème de santé publique. Ce travail s'inscrit donc dans le cadre de la politique d’établissement dont l'objectif est de déterminer les souches de bactéries isolées dans les urines des patients ainsi que leur sensibilité aux antibiotiques.

## Matériel et méthodes

Étude prospective menée chez les adultes hospitalisés pour infection du tractus urinaire au Centre hospitalier Marguerite de Lorraine. La répartition des espèces bactériennes et les profils de sensibilité ont été décrits.

## Résultats

Au total, 104 patients ont été inclus avec un sex-ratio de 1,47 en faveur des femmes. L’âge moyen était de 82,0 ± 12,8 ans [29-99 ans]. Les signes cliniques étaient dominés par la fièvre et/ou frissons (76 %), les signes fonctionnels urinaires (52,9 %) et l'hématurie (16 %). La quasi-totalité des patients (96 cas) présentait au moins un facteur de risque d'infection urinaire compliquée : âge avancé et fragilité chez 75 patients et insuffisance rénale chronique sévère chez 14 patients.

Cent dix (110) souches ont été isolées avec une prédominance d'entérobactéries (91/110), 5 patients avaient des souches de *Staphylococcus aureus. Escherichia coli* (68/110 souches) était majoritaire dont 14 souches étaient BLSE.

## Conclusion

Les entérobactéries dont *Escherichia coli* prédominent dans les infections du tractus urinaire au Centre hospitalier Marguerite de Lorraine. Une proportion non négligeable reste multirésistante. Le renforcement de la surveillance des bactéries multirésistantes et la mise en place d'un guide de prescription d'antibiotique semblent nécessaires pour limiter l’émergence des souches résistantes aux antibiotiques.

# Research article: Concentrations de l’éfavirenz dans le lait maternel et prédiction des quantités ingérées par le nourrisson allaité au Mali

**Published:** 2023-06-29

**Authors:** Aboubacar Alassane OUMAR, Kadiato BAGAYOKO-MAIGA, Aliou BAHACHIMI, Marie-Christine CERE, Étienne CHATELUT, Mariam SYLLA, Sounkalo DAO, Peggy GANDIA

**Affiliations:** 1 Laboratoire de pharmacocinétique et toxicologie, Institut f**édératif de** biologie (IFB), Université hospitalière Purpan, Toulouse, France; 2 Laboratoire de pharmacologie et pharmacogénétique EA4553, Institut universitaire du cancer Oncopole, Toulouse, France; 3 Centre de recherche et de formation VIH/TB, Université des sciences, des techniques et des technologies de Bamako, Mali; 4 Département de pédiatrie, CHU Gabriel Touré, Bamako, Mali

## Introduction

L'allaitement maternel augmente le risque de transmission du VIH de 14 %. Les données sont limitées sur la pharmacocinétique antirétrovirale dans le lait maternel. Dans ce travail, nous avons mesuré les concentrations plasmatiques et lactées des ARV des mères infectées par le VIH et de leurs nourrissons pendant l'allaitement.

## Matériel et méthodes

Les patients inclus étaient des femmes enceintes séropositives recevant une prophylaxie antirétrovirale de la semaine gestationnelle 25 jusqu’à 6 mois après l'accouchement et leurs nourrissons allaités. Des échantillons de sang ont été prélevés à l'accouchement et aux mois 1, 3 et 6 après l'accouchement. Les concentrations d’éfavirenz ont été mesurées par une méthode de chromatographie haute liquide en tandem avec la spectrométrie de masse. La limite de détection de la quantification était de 0,216 mg/l pour l’éfavirenz. La charge virale plasmatique a été mesurée sur M2000rt (Abbott). La limite de détection de la quantification virale était de 40 copies/ml. La charge virale a été déterminée à l'accouchement et à 6 mois post-partum pour les mères et à 3 et 6 mois post-partum pour les enfants. Tous les enfants ont reçu la névirapine pendant 6 semaines après la naissance.

## Résultats

Au total, 32 couples mère-enfant ont été inclus : 32 mères ont reçu du ténofovir (TDF), de la lamivudine (3TC) et de l’éfavirenz (EFV). Au cours de la grossesse, les mères ont reçu une combinaison de trois antirétroviraux qui a également été suivie pendant l'allaitement maternel (jusqu’à 6 mois) et par la suite. L’âge médian des mères était de 29 ans (19 à 40 ans). Le nombre moyen de cellules CD4+ à la livraison (cellules/mm^3^) était de 589,31. La concentration plasmatique maternelle médiane (IQR) en EFV était de 3105 ng/ml (2450-6595) au mois 1; 3050 ng/ ml (2060-6685) au mois 3; 2740 ng/ml (2150-8080) au cours du mois 6. La concentration plasmatique infantile médiane (IQR) en EFV était de 356 ng/ml (282-572,5) au mois 1; 268 ng/ml (142-464,5) au mois 3; et 175 ng/ ml (89,7-331) au mois 6. Le ratio médian (IQR) (plasma infantile/plasma maternel) était de 0,057 (0,031-0,1113) au mois 1; 0,072 (0,051-0,091) au mois 3 et 0,048 (0,033-0,070) au mois 6. Aucune réaction indésirable ou toxicité liée aux médicaments pris par la mère n'a été observée chez aucun des nourrissons. Une mère a présenté une charge virale > 50 copies/ ml à 6 mois, avec des concentrations plas-matiques du médicament indétectables à la même période.

## Conclusion

Les nourrissons allaités étaient exposés à de faibles quantités d'EFV, sans effet indésirable dans cette étude au Mali.

# Research article: Coût direct de la prise en charge initiale de l'hépatite virale B au Service des maladies infectieuses et tropicales de l'Hôpital national Donka

**Published:** 2023-06-29

**Authors:** Taïbou DIALLO, Kelety CAMARA, Aminata SYLLA, Ibrahima BAH, Mamadou Oury Safiatou DIALLO, Fodé Amara TRAORÉ, Fodé Bangaly SAKO, Mamadou Saliou SOW

**Affiliations:** 1 Service des maladies infectieuses et tropicales, Hôpital national Donka, CHU de Conakry, Guinée; 2 Faculté des sciences et techniques de la santé, Université Gamal Abdel Nasser de Conakry, Conakry, Guinée

## Introduction

L'infection par le VHB est un problème majeur de santé qui nécessite une prise en charge prolongée et très coûteuse. L'objectif était d’évaluer le coût direct de la prise en charge initiale de l'hépatite virale B au Service des maladies infectieuses et tropicales de l'Hôpital national Donka.

## Matériel et méthodes

Il s'est agi d'une étude transversale de type descriptif d'une durée de 6 mois allant du 2 mars au 31 août 2020. L'analyse a porté sur les aspects biologique et thérapeutique en termes de coût direct dans la prise en charge initiale des patients infectés par le virus de l'hépatite B.

## Résultats

Nous avons colligé 146 cas, toutes les couches socioprofessionnelles ont été atteintes, le sexe masculin était prédominant avec un sex-ratio de 58,9 %. La majorité des patients n'avaient pas d'assurance maladie (98 %) et venaient d'une zone urbaine (75,3 %). Le coût moyen des examens paracliniques était de 314 018,05 GNF (32,12 USD), celui du traitement était de 295 385 GNF (30,22 USD). Le coût moyen global de la prise en charge initiale de l'hépatite B était de 2 281 068,49 GNF (233,36 USD).

## Conclusion

Le coût de la prise en charge initiale de l'hépatite B est encore trop élevé en Guinée. Une prise en charge globale s'avère nécessaire, similaire à celle de l'infection à VIH.

# Research article: Délais de mise sous traitement ARV à l’ère du dépister-traiter : cas de l'Hôpital régional de Kankan

**Published:** 2023-06-29

**Authors:** Kadé FOFANA, Edgard Fassory TRAORÉ, Mamadou Diouldé KANTÉ, Mariama Bobo BAH, Mariama Saliou TOURÉ, Aissatou Bobo DIALLO, I. CAMARA, Mohamed Maciré SOUMAH, Keita MOUSSA, Thierno Mamadou TOUNKARA, Mohamed CISSÉ

**Affiliations:** 1 Service de dermatologie, Hôpital national Donka, CHU de Conakry, Guinée; 2 Faculté des sciences et techniques de la santé, Université Gamal Abdel Nasser de Conakry, Conakry, Guinée

## Introduction

Des avancées importantes ont été réalisées dans toute l'Afrique subsaharienne en ce qui concerne l'accessibilité des traitements antirétroviraux. Désormais, l'OMS recommande que le traitement ARV soit initié dans les 7 jours suivant le diagnostic du VIH et l’évaluation clinique, et que l'initiation du traitement ARV le jour même du diagnostic soit proposée aux patients. Notre étude avait donc pour but d’évaluer le délai de mise sous traitement ARV à l'Hôpital régional de Kankan.

## Matériel et méthodes

Il s'agissait d'une étude rétrospective à visée analytique d'une durée de trois (03) mois chez les patients dépistés et mis sous traitement ARV à l'Hôpital régional de Kankan entre janvier 2016 et décembre 2020.

## Résultats

Nous avons colligé 695 dossiers dont 634 ont répondu à nos critères d'inclusion. La tranche d’âge comprise entre 15 et 34 ans était la plus représentée, soit 41,9 % avec une prédominance féminine de 65,8 %; 65,3 % étaient des personnes mariées. 59,6 % résidaient en milieu urbain, 73,2 % étaient non scolarisés et 34,9 % étaient des femmes au foyer. La moitié ont été dépistés à un stade avancé de la maladie avec la présence d'infections opportunistes chez 57,5 %. Quant aux délais de mise sous traitement ARV après le dépistage, 66,7 % ont été mis sous traitement ARV dans le délai optimal; le délai moyen était de 68 jours. Les analyses uni- et multivariées n'ont pas révélé de déterminants à la mise sous traitement ARV.

## Conclusion

Les patients dépistés à l'Hôpital régional de Kankan ont débuté leur traitement ARV dans le délai optimal. La réalisation d'une étude à une plus large échelle pourrait identifier les leviers d'appui et les éventuels obstacles à la mise en œuvre des recommandations du dépister-traiter.

# Research article: Analyse comparative des techniques de criblage et de séquençage pour la détection des variants circulants du SARS-CoV-2 en Guinée

**Published:** 2023-06-29

**Authors:** Thibaut Armel Chérif GNIMADI, Haby DIALLO, Aminata MBAYE, Abdoul Karim SOUMAH, Kadio Jean-Jacques Olivier KADIO, Cécé KPAMOU, Abdoulaye TOURÉ, Alpha Kabinet KEITA

**Affiliations:** 1 Centre de recherche et de formation en infectiologie de Guinée (CERFIG), Conakry, Guinée; 2 Institut de recherche pour le développement, IRD/UMI233/INSERM-U1175, Université de Montpellier, Montpellier, France

## Contexte

Le test de PCR quantitative à transcription inverse (RT-qPCR) est la référence dans le diagnostic biologique de l'infection par le SARS-CoV-2. Il joue, avec le séquençage, un rôle majeur dans la détection et la surveillance des mutations importantes. Ces mutations sont le plus souvent à l'origine de l'apparition de nouveaux variants du virus. Nous avons comparé le test d'analyse des courbes de fusion (RT-qPCR/HRM) au séquençage Illumina pour la détection de mutations dans la glycoprotéine de pointe (Spike) du SARS-CoV-2 et avons examiné leur sensibilité et leur spécificité.

## Matériel et méthodes

Les analyses ont été réalisées au laboratoire de biologie moléculaire du Centre de recherche et de formation en infectiologie de Guinée dans le cadre du projet AFROSCREEN. Les échantillons positifs pour le SARS-CoV-2 avec un CT compris entre 15 et 28 cycles ont été criblés par la méthode d'analyse des courbes de fusion (RT-qPCR/HRM) en utilisant le Kit Innovative Diagnostic, ciblant les mutations N501Y, E484K/Q, K417N, L452R/Q et P681H/R. Puis le séquençage du génome entier a été effectué en utilisant la plateforme Illumina Iseq100. Les résultats des deux méthodes ont été comparés.

## Résultats

Cette comparaison porte sur 79 échantillons qui ont été criblés et séquencés. La méthode de criblage, qui cible certaines mutations de la protéine Spike, a permis d'identifier des variants pour 68/79 isolats. Onze (11) résultats étaient indéterminés.

Après séquençage, les séquences présentaient une bonne couverture > 80 %. Il ressort que sur les 68 échantillons détectés par le criblage, 65 correspondaient aux variants détectés par le séquençage. Trois isolats avaient des résultats non concordants avec les résultats obtenus au criblage.

L'accord entre le séquençage et le criblage était de 95,6 %. La sensibilité et la spécificité du criblage par rapport au séquençage étaient respectivement de 82,2 % et 100 %.

## Conclusion

L'accord, la sensibilité, la spécificité et le délai d'exécution du criblage sont bien adaptés à la surveillance génomique du SARS-CoV-2, ce qui en fait un outil important en période de pic.

# Research article: Dermohypodermites bactériennes chez les enfants de 0 à 18 ans : aspects épidémiologiques, cliniques, thérapeutiques et évolutifs au Service de dermatologie-vénéréologie de l'Hôpital national Donka

**Published:** 2023-06-29

**Authors:** Mariama Bobo BAH, Priska Carine Dibahga WANDJ, Aissatou Bobo DIALLO, Mamadou Diouldé KANTÉ, Mariama Saliou TOURÉ, Mamadou Bademba BARRY, Fatoumata KEITA, Diané BOH FANTA, Mohamed Maciré SOUMAH, Moussa KEITA, Fodé Bangaly SAKO, Thierno Mamadou TOUNKARA

**Affiliations:** 1 Service de dermatologie, Hôpital national Donka, CHU de Conakry, Guinée; 2 Faculté des sciences et techniques de la santé, Université Gamal Abdel Nasser de Conakry, Conakry, Guinée; 3 Service des maladies infectieuses et tropicales, CHU de Conakry, Guinée

## Introduction

Les dermohypodermites bactériennes (DHB) sont des infections bactériennes aiguës nécrosantes ou non des tissus cutanés dues soit au streptocoque β-hémolytique du groupe A (érysipèle) soit aux germes polymicrobiens (DHBN-FN). Le but de ce travail était d’étudier les aspects épidémiologiques, cliniques, thérapeutiques et évolutifs des DHB chez les enfants au Service de der-matologie-vénéréologie de l'Hôpital national Donka.

## Matériel et méthodes

Étude rétrospective de type descriptif d'une durée de 6 ans, tous les enfants âgés de 0 à 18 ans ayant développé une DHB ont été inclus.

## Résultats

Quatre-vingt-seize dossiers étaient colligés en 6 ans soit une prévalence de 2 %. L’âge moyen était de 12,59 ans avec des extrêmes de 2 ans et 18 ans. Le sex-ratio H/F était de 0,92. Le délai moyen d’évolution des symptômes était de 8,22 jours. Les facteurs de risque étaient l'utilisation d'AINS dans 44 cas, la phytothérapie à base de cataplasmes dans 42 cas, la dépigmentation dans 10 cas. Les formes cliniques étaient non nécrosantes dans 57 cas et nécrosantes dans 39 cas. Les lésions étaient localisées aux membres inférieurs dans 90 cas, aux membres supérieurs dans 2 cas, et au visage dans 4 cas. Le traitement médical était à base d'antibiotiques chez tous les patients, associé à une chirurgie par nécrosectomie dans 39 cas. L’évolution était favorable dans 94 cas. Nous avons enregistré 2 décès par sepsis parmi les cas de fasciite nécrosante.

## Conclusion

Notre travail confirme la rareté des DHB chez les enfants. Elles sont dominées par les formes non nécrosantes. Cependant, la surveillance doit être rigoureuse afin de ne pas méconnaître les formes nécrosantes.

# Research article: Étude de l'utilisation des stratégies de prévention du VIH chez les hommes ayant des rapports sexuels avec les hommes (HSH) au Sénégal

**Published:** 2023-06-29

**Authors:** Alpha Mamadou DIALLO, Mbaye Khardiata DIALLO, Viviane Marie Pierre CISSÉ, Ndeye Aissatou LAKHE, Daye KA, Ndeye Maguette FALL, Thioub DAOUDA, Aboubakar Sidikh BADIANE, Latyr Junior DIOUF, Alassane SARR, Louise FORTES, Cheikh Tidiane NDOUR, Masserigne SOUMARÉ, Seydi MOUSSA

**Affiliations:** 1 Service des maladies infectieuses, Centre hospitalier national universitaire de Fann, Dakar, Sénégal; 2 Division Lutte contre le sida et les IST au Sénégal

## Contexte

Les HSH constituent un groupe de la population particulièrement affecté par l'infection par le VIH. Plusieurs interventions sont mises en œuvre au profit de cette population. Elles visent à produire un changement de comportement, mais aussi à réduire le fardeau de la maladie. En 2017, la Division Lutte contre le sida et les IST (DLSI), en collaboration avec le Comité national de lutte contre le sida (CNLS) et les partenaires au développement, avait initié une enquête afin de documenter l'effet de ces interventions.

## Objectifs

Étudier les déterminants du recours aux mesures et stratégies de prévention du VIH chez les HSH au Sénégal, évaluer leurs connaissances par rapport au VIH/sida, évaluer la fréquence et les circonstances de leur utilisation du préservatif, et enfin, déterminer leurs comportements ayant un impact sur les risques d'infection.

## Méthodologie

Enquête transversale descriptive conduite en 2017 au niveau de 12 régions du Sénégal. Ont été inclus dans cette étude les adultes de sexe masculin, d’âge supérieur à 18 ans, admettant avoir des relations sexuelles avec d'autres hommes, ayant donné un consentement libre et éclairé.

## Résultats

Au total, 1148 HSH ont participé à l'enquête dont 31 % à la capitale. L’âge médian était de 24 ans. La majorité présentait un profil bisexuel (77 %), célibataire (91 %), instruit jusqu'au niveau secondaire à 51 %, et 36 % des HSH avaient déjà eu un rapport homosexuel entre l’âge de 15 à 17 ans. Ils ont été 59 % au moins à être touchés par des actions de sensibilisation. La pénétration non protégée était le principal mode de transmission du VIH cité à 21 %, et 84 % étaient au courant de l'existence de structures de dépistage du VIH. L'existence d'un lieu de prise en charge pour HSH était connue par 11 % de la population d’étude; seuls 7 % d'entre eux ont au moins une fois fréquenté ces lieux de prise en charge des HSH. L'existence d'un traitement ARV était connue par 5 % d'entre eux. La multiplicité des partenaires sexuels était notée (jusqu’à dix) avec une utilisation systématique de préservatif au cours de rapports anaux passifs et actifs respectivement à 74 % et 73 %. La prévalence du VIH était estimée à 27,6 %, dont la moitié vivait à Dakar et 46 % étaient des sujets âgés de 25 ans et plus.

## Conclusion

Pour réduire la prévalence du VIH au sein de ce groupe où un HSH sur quatre est infecté par le VIH, des programmes de prévention et de prise en charge spécifiques adaptés à leurs besoins s'avèrent nécessaires dans notre pays.

# Research article: Évaluation de la biosécurité et facteurs associés à l'influenza aviaire dans les fermes avicoles de Coyah, Guinée, 2019-2020

**Published:** 2023-06-29

**Authors:** Émile Faya BONGONO, Lancei KABA, Alioune CAMARA, Abdoulaye TOURÉ, Mavungu Patrick NGOMA, Pauline Kiswensida YANOGO, Estelle KANYALA, Adama SOW

**Affiliations:** 1 Burkina Field Epidemiology and Laboratory Training Program, Université Joseph Ki-Zerbo, Ouagadougou, Burkina Faso; 2 Institut supérieur des sciences et de médecine vétérinaire (ISSMV), Dalaba, Guinée; 3 Centre de recherche et de formation en infectiologie de Guinée (CERFIG), Campus Hadja Mafory Bangoura, Université Gamal Abdel Nasser de Conakry, Guinée; 4 African Epidemiology Network (AFENET), Conakry, Guinée; 5 Organisation des Nations unies pour l'alimentation et l'agriculture (FAO), Ouagadougou, Burkina Faso; 6 Organisation des Nations unies pour l'alimentation et l'agriculture (FAO), Coléah, Guinée

## Introduction

La biosécurité est un problème de santé publique. Elle permet de réduire le risque d'introduction et la propagation de la maladie, et de diminuer la morbidité et la mortalité. L'objectif de cette étude était d’évaluer le niveau de biosécurité et d'identifier le risque de survenue de l'influenza aviaire dans les fermes avicoles de Coyah en Guinée.

## Matériel et méthodes

Il s'agissait d'une étude transversale réalisée du 18 décembre 2019 au 27 mars 2020 dans les fermes avicoles de Coyah. Un questionnaire semi-structuré a été utilisé pour la récolte de données sur la biosécurité. Ainsi, à l’échelle de l’élevage on distingue la biosécurité externe qui vise à empêcher l'introduction de nouveaux agents infectieux dans une ferme, et la biosécurité interne qui vise à réduire la propagation de la maladie à l'intérieur de l’élevage. Ensuite l'outil « Biocheck.UGent » a été utilisé pour apprécier cette biosécurité. En analyse uni- et multivariée, nous avons utilisé une régression logistique pour calculer les OR d'association.

## Résultats

Au total, 84 (100 %>) fermes étaient évaluées avec un faible niveau de biosécurité interne. Cependant, 55 (65,48 %) et 29 (34,52 %) fermes avaient respectivement un niveau de biosécurité externe faible et moyen. Les facteurs de risque associés à l'influenza aviaire étaient : les cadavres d'oiseaux à l'intérieur et à l'extérieur des bâtiments d’élevage respectivement (OR = 55,33 [3,72-823,93]; p = 0,004 et 6,66 [1,25-35,48]; p = 0,026) et la présence d'oiseaux sauvages et domestiques. Le nettoyage du sol et des parois a cependant été un facteur protecteur (OR = 0,13 [0,02-0,69]; p = 0,017).

## Conclusion

La biosécurité reste une pratique préventive dans la lutte contre les maladies. La prise en compte des résultats de cette étude permettra d'améliorer la grille d’évaluation de la biosécurité dans les fermes avicoles.

# Research article: Facteurs prédictifs de décès chez les patients tuberculeux monorésistants à la rifampicine traités dans les centres de traitement antituberculeux de Conakry

**Published:** 2023-06-29

**Authors:** Salifou Talassone BANGOURA, Boubacar Djelo DIALLO, Alioune CAMARA, Philippe VANHEMS, Aboubacar Sidiki MAGASSOUBA, Saidouba Cherif CAMARA, Maladho DIABY, Kadio Jean-Jacques Olivier KADIO, Mamadou Saliou SOW, Nagham KHANAFER, Abdoulaye TOURÉ

**Affiliations:** 1 Centre de recherche et de formation en infectiologie de Guinée (CERFIG), Campus Hadja Mafory Bangoura, Université Gamal Abdel Nasser de Conakry, Guinée; 2 Chaire de santé publique, Université Gamal Abdel Nasser de Conakry, Guinée; 3 Programme national de lutte antituberculeuse, Conakry, Guinée; 4 Service de pneumo-phtisiologie, Hôpital national Ignace Deen, Conakry, Guinée; 5 Service des maladies infectieuses et tropicales, Hôpital national Donka, Conakry, Guinée; 6 Santé publique, épidémiologie et écologie évolutive des maladies infectieuses, Centre international de recherche en infectiologie (CIRI), INSERM-U1111-UCBL Lyon 1-ENS Lyon, France; 7 Service Hygiène hospitalière, *é*pidémiologie et prévention, Hôpital *Édouard* Herriot, Hospices civils de Lyon, Lyon, France

## Contexte

L’émergence de la tuberculose multirésistante (TB-MR) constitue un problème majeur pour les programmes de lutte contre la tuberculose en raison du risque élevé de décès et d’échec thérapeutique. L'objectif de cette étude était d'analyser la survie et les facteurs prédictifs de décès chez les patients TB-MR traités avec le régime court (9-11 mois) entre 2016 et 2021 dans les centres de traitement antituberculeux de Conakry.

## Méthodes

Les données collectées prospec-tivement entre 2016 et 2021 dans le Service de pneumo-phtisiologie de l'Hôpital national Ignace Deen et dans les centres antituberculeux de la Carrière et Tombolia ont été analysées. La méthode de Kaplan Meier, le test de log-Rank et le modèle de Cox ont été utilisés respectivement pour la construction, la comparaison des courbes de survie et l'identification des facteurs prédictifs de décès. L'hypothèse de risque proportionnel a été vérifiée par le test de résidus de Schönefeld.

## Résultats

Sur un total de 1075 patients TBMR, les données de 869 patients ont été analysées. Parmi ceux-ci, 164 (18,9 %) patients sont décédés pendant le traitement dont 126 au cours des 120 jours suivant le début du traitement. La survie globale à 11 mois était de 79 %. Indépendamment des autres facteurs, les patients traités dans le Service de pneumo-phtisiologie de l'Hôpital Ignace Deen (HRa^1^1Hazard Risk ajusté soit Rapport de risque ajusté (RRa). = 2,90; IC95% [1,88-4,46]), ceux âgés de plus de 45 ans (HRa = 2,61; IC95% [1,61-4,22]) et ceux co-infectés par le VIH (HRa = 2,76; IC95% [1,90-4,00]) étaient plus à risque de décéder pendant le traitement. Cependant, les patients avec un antécédent de traitement antituberculeux de 1^re^ ligne avaient un risque de décès réduit de 32 % (HRa = 0,68; IC95% [0,48-0,98]).

## Conclusion

Ces résultats soulignent la nécessité de garantir davantage le diagnostic précoce, l'usage rationnel des antituberculeux ainsi que l'observance du traitement afin d'améliorer la survie des patients.

# Research article: Incidence et facteurs associés à la perte de suivi des patients sous thérapie antirétrovirale au Centre de santé de Gbessia Port 1, Conakry

**Published:** 2023-06-29

**Authors:** Foromo GUILAVOGUI, Niouma Nestor LENO, Thierno Souleymane DIALLO, Pe DAMEY, Jean-Michel LAMAH, Alexandre DELAMOU, Timothée GUILAVOGUI

**Affiliations:** 1 Faculté des sciences et techniques de la santé, Université Gamal Abdel Nasser de Conakry, Conakry, Guinée; 2 Programme national de lutte contre le VIH/sida et les hépatites, Conakry, Guinée

## Introduction

Cette étude visait à déterminer l'incidence cumulée des perdus de vue (PDV), estimer le taux de survie des patients suivis et identifier les facteurs associés à la perte de suivi des patients sous antirétroviraux (ARV).

## Matériel et méthodes

Il s'agissait d'une cohorte rétrospective réalisée au Centre de santé urbain de Gbessia Port 1 à Conakry de 2014 à 2020, portant sur les patients âgés de 15 ans ou plus ayant initié le traitement antirétroviral (TAR) il y a au moins 6 mois. Le logiciel SPSS, les courbes de Kaplan Meier et le modèle derégression logistique multivariée ont servi respectivement pour l'analyse des données, l'estimation de la rétention et l'identification des facteurs associés.

## Résultats

L'incidence cumulée des perdus de vue était à 16,5 % en 24 mois. La probabilité pour qu'un patient soit perdu de vue était respectivement de 5 % à 6 mois, 25 % à 12 mois, 40 % à 24 mois, 59 % à 36 mois et 70 % à 48 mois. Le taux de survie des patients sous ARV suivis variait respectivement de 94,3 % à 6 mois avec un IC95% de [93,8-96,1]; 86,7 % à 12 mois avec un IC95% de [76,7-90,7]; 73,3 % à 24 mois avec un IC95% de [69,8-84,8] et 62,1 % à 48 mois (4 ans) avec un IC95% de [53,9-71,4]. Le niveau d'instruction, le stade I de l'OMS et la charge virale de base < 1000 copies/ml étaient associés à la perte de vue des patients.

## Conclusion

Le taux d'incidence de PDV augmente avec le nombre d'années suivant un TAR, et la rétention des patients à 4 ans est bien inférieure à l'objectif d'au moins 70 % fixé dans le Cadre stratégique national sur le VIH et les IST (2018-2022).

# Research article: Manifestations oculaires liées à l'infection au VIH au Centre de traitement ambulatoire et de prise en charge du CHU de Donka à Conakry en Guinée du 1^er^ mars au 31 août 2020

**Published:** 2023-06-29

**Authors:** Christophe ZOUMANIGUI, Maxime Dantouma SOVOGUI, Abdoul Karim BALDE, Jeanette Gbawi BILIVOGUI, Kokou VONOR

**Affiliations:** 1 Faculté des sciences et techniques de la santé, Université Gamal Abdel Nasser de Conakry, Conakry, Guinée; 2 Clinique ophtalmologique Bartimée, Conakry, Guinée; 3 Centre hospitalo-universitaire Donka, Conakry, Guinée; 4 Centre hospitalier régional de Kara, Togo

## Objectif

Identifier les lésions ophtalmologiques observées au cours de l'infection à VIH/sida.

## Méthodologie

Il s'agit d'une étude transversale descriptive et analytique de 6 mois. Le recrutement était exhaustif et les considérations éthiques étaient respectées. Les données ont été collectées à l'aide d'un questionnaire structuré en français. La première partie du questionnaire comprenait des données démographiques; la deuxième, les résultats de l'examen ophtalmologique et le bilan biologique. Nos variables étaient épidémiologiques, cliniques et biologiques et p < 0,05 était considéré comme statistiquement significatif.

## Résultats

Nous avons rapporté une fréquence de 73,14 % soit 128 patients. Au total, 365 lésions ophtalmologiques ont été recensées, dont 85 soit 23,29 % au niveau des annexes, 146 soit 40 % au niveau du segment antérieur et 134 soit 36,71 % au niveau du segment postérieur. Les lésions les plus fréquentes étaient les conjonctivites infectieuses 53 cas soit 14,52 %, l'uvéite antérieure 67 cas soit 18,36 %, les kératites 59 cas soit 16,16 %, l'hyalite 36 cas soit 9,86 %, les hémorragies rétiniennes 32 cas soit 8,78 %, les nodules cotonneux 14 cas soit 3,84 %, les vascularites/périvascularites 12 cas soit 3,29 %, la choriorétinite et l'ischémie rétinienne 11 cas chacun soit 3,01 %. Le taux moyen de CD4 dans la population d’étude était de 208 ± 143,3/mm^3^, dont 52,57 % soit 92 patients avaient un taux < 200/mm^3^ et 47,43 % soit 83 patients avaient un taux ≥ 200/ mm^3^. Il existait un lien statistique entre la survenue des lésions rétiniennes (nodules cotonneux, hémorragie, vascularites, ischémie, œdème papillaire) avec la sévérité de l'immunodépression, CD4 < 200/mm^3^ soit p < 0,05.

## Conclusion

La prévalence des atteintes ophtalmologiques est élevée chez les PvVIH à Conakry. Elles sont d'autant plus fréquentes que le taux de CD4 est bas. Les lésions du segment antérieur sont les plus fréquentes. Toutefois, la fréquence élevée des atteintes rétiniennes nous interpelle.

# Research article: Neuro-tuberculose : à propos de 16 cas consécutifs au Service de neurologie de l'Hôpital national Ignace Deen

**Published:** 2023-06-29

**Authors:** Malé DORÉ, Souleymane Djigué BARRY, Mohamed Lamine TOURÉ, Mohamed Lelouma MANSARÉ, Kaba CONDÉ, Mohamed Lamine CONDÉ, Karinka DIAWARA, Adama KONÉ, Idrissa DOUMBOUYA, Mamady CAMARA, Issiaga BAH, Fodé Abass CISSÉ

**Affiliations:** 1 Service de neurologie, Hôpital national Ignace Deen, Conakry, Guinée; 2 Service de neurologie, Hôpital national Donka, Conakry, Guinée

## Introduction

La neuro-tuberculose (NTB) est une complication rare de la tuberculose pulmonaire et la plus grave; elle reste sous-estimée dans notre contexte. L'objectif de ce travail était de décrire les profils épidémiologiques, cliniques, paracliniques et évolutifs de la NTB.

## Matériel et méthodes

Nous avons effectué une étude rétrospective d'une durée d'un an allant du 1^er^ juillet 2019 au 30 juin 2020 portant sur les dossiers médicaux des patients atteints de neuro-tuberculose. Nos variables ont été qualitatives et quantitatives, réparties en données épidémiologiques, cliniques, thérapeutiques et évolutives.

## Résultats

Au cours de cette étude, 500 patients ont été hospitalisés parmi lesquels 16 patients avaient une neuro-tuberculose soient une fréquence de 3,2 %. L’âge moyen des patients était de 29,5 ± 13,7 ans avec une prédominance masculine (sex-ratio : 1,7). L'infection par le VIH et l'antécédent de tuberculose pulmonaire étaient majoritairement représentés avec 37,5 %. À l'examen clinique, la fièvre était présente chez tous nos patients. Un déficit neurologique focal a été retrouvé chez 13 patients (81,2 %), suivi d'un syndrome rachidien dans 43,8 % des cas. L'intrader-moréaction à la tuberculine ≥ 10 mm était présente chez tous nos patients. À l'analyse du LCS, l'hyperprotéinorachie et la pléiocytose étaient retrouvées dans respectivement 80,5 % et 75,2 % des cas. L'hypoglycorachie était retrouvée chez 50 % des patients. La tuberculose vertébrale a été la plus représentée chez 50 % des patients, suivie de la méningo-encéphalite chez 25 % des patients. Tous nos patients ont bénéficié d'un traitement antituberculeux, et l’évolution a été favorable chez 80 % des patients sous traitement antituberculeux.

## Conclusion

La NTB est une maladie rare mais avec des signes et symptômes non spécifiques. Le diagnostic peut être difficile. Elle doit être considérée comme un diagnostic différentiel chez les patients présentant une affection du système nerveux central dans notre contexte.

# Research article: Prévalence de l'hépatite B chez les patients atteints du VIH au Centre hospitalier régional spécialisé de Macenta

**Published:** 2023-06-29

**Authors:** Aubin ZOUMANIGUI, Fodé Bangaly MAGASSOUBA, Alimou CAMARA, Sanaba BOMBALY

**Affiliations:** Centre de recherche en virologie (CRV), Guinée

## Introduction

Le virus de l'immunodéficience humaine (VIH) et celui de l'hépatite B provoquent de véritables problèmes de santé publique dans les pays tropicaux. L'objectif de cette étude était de déterminer la prévalence de l'hépatite B chez les patients vivant avec le VIH (PvVIH) au Centre hospitalier régional spécialisé de Macenta.

## Méthodes

Il s'agissait d'une étude rétrospective de type descriptif d'une période de 6 ans allant du 1^er^ janvier 2015 au 31 décembre 2020. L’étude portait sur tous les dossiers PvVIH soumis au diagnostic de l'antigène HBs durant la période d’étude. Nos variables étaient quantitatives et qualitatives.

## Résultats

Sur un total de 1020 personnes vivant avec le VIH, 75 étaient porteuses du virus de l'hépatite B soit une prévalence globale de 7,35 % [5,75-8,95]. L’âge moyen de nos patients était de 39,5 ans (plus ou moins 5 ans) avec un sex-ratio de 0,77. La répartition selon le genre montrait une prédominance féminine, soit 56,47 %. Les ménagères constituaient la couche socioprofessionnelle la plus représentée, soit 30,49 %. La tranche d’âge de 28-35 ans était la plus atteinte par l'hépatite B, soit 1,96 %. Les mariés étaient plus touchés, soit 5,00 %.

## Conclusion

Le VIH/sida et l'hépatite B causent de sérieux problèmes de santé publique, en particulier dans les pays en développement. Notre étude a montré une prévalence de l'hépatite B relativement élevée chez les PvVIH. Ceci mérite d’être pris au sérieux afin de mettre des stratégies en place pour lutter contre ces deux pathologies chroniques.

# Research article: Tuberculose neuroméningée : aspects épidémiologiques, cliniques, paracliniques et évolutifs au Service des maladies infectieuses du Centre hospitalier national et universitaire (CHNU) de Fann à Dakar, Sénégal

**Published:** 2023-06-29

**Authors:** Viviane Marie Pierre CISSÉ, Daouda THIOUB, Walimata HANE, Mbaye Khardiata DIALLO, Ndeye Aissatou LAKHE, Ndeye Maguette FALL, Aboubakar Sidikh BADIANE, Latyr Junior DIOUF, Alassane SARR, Daye KA, Assane DIOUF, Louise FORTES, Moussa SEYDI

**Affiliations:** Service des maladies infectieuses et tropicales Ibrahima Diop Mar, Centre hospitalier national universitaire de Fann, Dakar, Sénégal

## Introduction

La tuberculose neuroméningée demeure un problème de santé publique dans les pays à ressources limitées. Le grand polymorphisme clinique et le manque de spécificité des signes radiologiques rendent le diagnostic difficile et sont fréquemment responsables d'un retard de prise en charge. Nous avons mené une étude sur les aspects épidémiologiques, cliniques, paracliniques et évolutifs de la tuberculose neuroméningée dans le Service des maladies infectieuses et tropicales du CHNU de Fann à Dakar.

## Matériel et méthodes

Il s'agit d'une étude rétrospective à visée descriptive et analytique portant sur les patients atteints de tuberculose neuroméningée durant la période allant de janvier 2015 à décembre 2020

## Résultats

Durant notre étude, 55 patients ont été hospitalisés pour une tuberculose neuroméningée soit une prévalence de 1,03 %. La prédominance masculine était nette avec un sex-ratio de 3,23, un âge moyen de 40 ans, d'un niveau socio-économique faible dans 34,55 % des cas. La notion de contage a été retrouvée chez 14,5 %, une tuberculose antérieure chez 25 % et la séroprévalence du VIH était de 41,82 %. Les céphalées et la fièvre étaient les plus fréquentes. Les troubles de conscience ont été retrouvés dans 63,64 %, les troubles de comportement 29,09 % à type d'agitation psychomotrice, les crises convulsives dans 18,18 %, les signes méningés dans 95,55 %, l'hypertension intracrânienne dans 56,36 %, une paralysie des nerfs crâniens dans 38,18 %. Le liquide cérébro-spinal (LCS) était clair chez 73,47 % avec une prédominance lymphocy-taire chez 71,4 %. Une hypoglycorachie a été retrouvée chez 71,4 % et une hyperprotéinorachie chez 77,5 % des cas. Le GeneXpert dans le LCS était positif chez 9,09 %. La tomoden-sitométrie (TDM) cérébrale retrouvait un tuberculome chez 23,36 %. Tous les patients ont bénéficié d'un traitement anti-tuberculeux (2RHZE/10RH). Le délai de début du traitement était en moyenne de 6,1 jours. Des complications étaient survenues chez 69,8 %, et 21,8 % des cas étaient décédés.

## Conclusion

La tuberculose neuroméningée est une urgence diagnostique et thérapeutique. Il faut y penser dans notre contexte devant un tableau de fièvre subaiguë ou prolongée associée à des signes neurologiques.

# Research article: Caractéristiques épidémiolo-giques, cliniques, paracliniques et évolutives des patients admis pour paludisme grave au Service des maladies infectieuses et tropicales du CHNU de Fann à Dakar, Sénégal, de 2019 à 2021

**Published:** 2023-06-29

**Authors:** Ibrahima Sory SOUMAH, Viviane Marie Pierre CISSÉ, Mamadou SECK, Malado DIALLO, Aboubakar Sidikh BADIANE, Alassane SARR, Latyr Junior DIOUF, Daouda THIOUB, Ndeye Maguette FALL, Mbaye Khardiata DIALLO, Ndeye Aissatou LAKHE, Daye KA, Assane DIOUF, Louise FORTES, Sylvie Audrey DIOP, Cheikh Tidiane NDOUR, Masserigne SOUMARÉ, Moussa SEYDI

**Affiliations:** 1 Service des maladies infectieuses et tropicales Ibrahima Diop Mar, Centre hospitalier national universitaire de Fann, Dakar, Sénégal; 2 UFR des sciences de la santé, Université de Thiès, Thiès, Sénégal

## Introduction

L'amélioration des stratégies de prévention et le diagnostic précoce avec l'avènement du test de diagnostic rapide (TDR) ont entraîné une diminution des cas de paludisme grave, cependant il demeure un problème de santé publique dans les pays à ressources limitées.

## Patients et méthodes

Il s'agit d'une étude rétrospective sur les aspects épidémiologiques, cliniques, paracliniques et évolutifs des cas de paludisme grave hospitalisés au Service de maladies infectieuses et tropicales du CHNU de Fann durant la période allant de janvier 2019 à décembre 2021.

## Résultats

Durant notre étude, 138 patients ont été hospitalisés pour paludisme grave sur une période de 3 ans avec un pic au mois d'octobre et de novembre. Ils provenaient le plus souvent des districts centre (24,4 %), sud (20,6 %) et nord (19,1 %). Le délai moyen entre le début de la symptomatologie et l'admission était de 6,7 jours ± 7,1; chez 18 % des patients ce délai était supérieur à 7 jours. La prédominance masculine était nette avec un sex-ratio de 3,18, la moyenne d’âge des patients était de 33,9 ans ± 17 ans dont 76 % entre 16 et 45 ans. Les principaux signes de gravité clinique étaient représentés par le coma (47 %), la prostration (37,8 %) et les convulsions (7,4 %). La fièvre était retrouvée chez 93,3 %. Sur le plan biologique on retrouvait majoritairement l'anémie (14,5 %) et l'insuffisance rénale chez 9,4 %. La protéine C-réactive était positive dans 96,3 % des cas. Le paludisme grave était associé à l'infection au SARS-CoV-2 chez 4 patients et à la dengue chez 1 patient.

Tous les patients ont bénéficié d'un traitement à base d'artésunate par voie injectable associé à une antibiothérapie chez 22 patients. La durée moyenne d'hospitalisation était de 5 jours. La létalité était de 10,7 %.

## Conclusion

Le paludisme grave demeure un problème de santé publique dans nos pays à ressources limitées. L'accès au TDR a amélioré le diagnostic rapide. Un renforcement des mesures de prévention et une prise en charge précoce peuvent améliorer le pronostic des patients.

# Research article: Risque rabique chez les personnes mordues par les animaux à sang chaud : aspects épidémiologique et prophylactique au Centre de traitement des épidémies de l'Hôpital régional de Kindia, Guinée

**Published:** 2023-06-29

**Authors:** Mamadou Oury Safiatou DIALLO, Ibrahima FOFANA, Fodé Amara TRAORÉ, Ibrahima BAH, Karamba SYLLA, Fodé Bangaly SAKO, Mamadou Saliou SOW

**Affiliations:** Service des maladies infectieuses et tropicales, Hôpital national Donka, CHU de Conakry, Chaire de dermatologie et des maladies infectieuses, Université Gamal Abdel Nasser de Conakry, Guinée

## Introduction

La prise en charge des personnes mordues par les animaux à sang chaud obéit à des attitudes stéréotypées et impose un traitement médical et chirurgical en urgence, seul susceptible d'enrayer une abcédation. L'objectif de cette étude était de décrire les modalités de prise en charge du risque rabique au Centre de traitement des épidémies (CT-Épi) de l'hôpital régional de Kindia chez les personnes qui consultent après une exposition au risque de rage.

## Matériel et méthodes

Il s'agissait d'une étude rétrospective de type descriptif allant du 1^er^ janvier 2014 à octobre 2019.

## Résultats

Durant la période d’étude, 415 patients exposés au risque rabique ont été colligés au Centre de traitement des épidémies de l'Hôpital régional de Kindia. L’âge moyen était de 25 ans avec les extrêmes de 1 et 90 ans et un sex-ratio de 1,18. La majorité (82,65 %) des cas provenait de la préfecture de Kindia. Le chien était l'animal mordeur le plus retrouvé dans 381 (91,81 %) des cas, suivi du chat dans 29 (6,99 %) des cas. La morsure a été le motif de consultation le plus fréquent avec 91,81 %. Le pied était le siège de la morsure dans 84,34 % des cas. La grande proportion des sujets exposés (42,89 %) ont été pris en charge dans les 24 heures qui ont suivi leur exposition. La quasi-totalité des patients ont bénéficié d'une vaccination à l'aide du protocole de Zagreb.

## Conclusion

Le risque rabique reste toujours d'actualité et sa prise en charge passe par les soins locaux et l'administration de vaccin anti-rabique.

La vaccination systématique des chiens reste le moyen le plus efficace pour réduire le risque rabique dans la région administrative de Kindia.

# Research article: La cryptococcose neuroméningée et l'infection au VIH dans le Service des maladies infectieuses et tropicales de l'Hôpital national Donka

**Published:** 2023-06-29

**Authors:** Lansana CAMARA, Aguibou SOW, Karamba SYLLA, Kelety CAMARA, Aminata SYLLA, Ibrahima BAH, Mamadou Oury Safiatou DIALLO, Fodé Amara TRAORÉ, Fodé Bangaly SAKO, Mamadou Saliou SOW

**Affiliations:** 1 Service des maladies infectieuses et tropicales, Hôpital national Donka, CHU de Conakry, Guinée; 2 Faculté des sciences et techniques de la santé, Université Gamal Abdel Nasser de Conakry, Guinée

## Introduction

La cryptococcose neuroméningée est la deuxième affection opportuniste du système nerveux rencontrée au cours du VIH/sida en Afrique subsaharienne. L'objectif était de décrire les aspects épidémiologiques, cliniques, biologiques et évolutifs de la cryp-tococcose neuroméningée.

## Matériel et méthodes

Il s'agissait d'une étude rétrospective et descriptive menée à partir des dossiers médicaux des patients hospitalisés pendant 6 ans allant du 1^er^ janvier 2016 au 31 décembre 2021 dans le Service des maladies infectieuses et tropicales à l'Hôpital national Donka.

## Résultats

42 cas de cryptococcose neuroméningée (3,1 %) ont été diagnostiqués sur un total de 1397 patients hospitalisés. La majorité était de sexe masculin soit 54,8 %, l’âge moyen des patients était de 41,94 ans ± 8,99 avec des extrêmes de 26 et 58 ans. 61,9 % des patients étaient mariés. Les 42 patients étaient tous infectés par le VIH de type 1 et étaient au stade IV de la classification de l'OMS. Les manifestations cliniques les plus représentées étaient les céphalées (95,2 %) et la fièvre (83,3 %). À 88,1 % le liquide céphalo-rachidien était clair avec une prédominance lymphocytaire. L'Ag cryptococcique était positif dans 95,2 % des cas. La moyenne des lymphocytes CD4 était de 83,69/mm^3^ ± 89,88. Tous les patients étaient sous fluconazole soit 100 %; le délai de séjour moyen était de 15,29 jours ± 3,22 avec des extrêmes de 1 et 45 jours. 64,3 % des patients étaient sortis guéris.

## Conclusion

La réduction de la mortalité par cette mycose impose la nécessité d'un diagnostic rapide et d'un traitement approprié par le respect de l'utilisation du protocole recommandé.

# Research article: Profil des affections neurochirurgicales à l'Hôpital ANAIM de Kamsar

**Published:** 2023-06-29

**Authors:** Mohamed CHERIF, Mory CAMARA, Mawugnon Éric KOUDJO, Élisabeth TRAORÉ, Mohamed Lamine SYLLA, Ibrahima Sory SOUARÉ

**Affiliations:** Service de neurochirurgie, Hôpital de l'amitié sinoguinéenne (HASIGUI), Kipé, Université Gamal Abdel Nasser de Conakry, Guinée

## Introduction

L'avènement du scanner à l'Hôpital de l'ANAIM (Agence nationale d'aménagement des infrastructures minières) offre de nouvelles possibilités dans le diagnostic des pathologies du système nerveux central (SNC). L'objectif était de décrire le profil des pathologies neurochirurgicales prises en charge dans le contexte de précision diagnostique.

## Matériel et méthodes

Il s'agit d'une étude rétro-prospective de type descriptif de trois ans et trois mois allant du 1^er^ novembre 2018 au 31 janvier 2022.

## Résultats

Nous avons colligé 295 cas d'affections neurochirurgicales dans 3 services de l'Hôpital ANAIM de Kamsar dont 283 ont été inclus dans l’étude. La tranche d’âge de 31-69 ans était la plus représentée (21,2 %). Le sexe masculin était le plus représenté. Les signes d'atteintes cérébrales les plus retrouvés étaient les céphalées (44,17 %) suivies de la notion de perte de connaissance initiale (PCI) dans 25,8 % des cas. Parmi les atteintes rachidiennes, la symptomatologie était dominée par la lombalgie (14,8 %) et le signe de la sonnette (13,1 %). Parmi les irritations radiculaires, le signe de Lasègue (9,2 %) était le plus représenté. Les lésions du scanner cranio-encéphalique les plus retrouvées étaient les contusions cérébrales (5,7 %). La hernie discale (HD) paramédiane (3,9 %) était la lésion la plus fréquente au scanner rachidien. La lésion la plus retrouvée à la radio standard du rachis était le pincement discal (1,8 %). L'IRM a permis le diagnostic des HD dans 2,1 % des cas. La pathologie traumatique (66,4 %) suivie de la pathologie dégénérative (14,1 %) étaient les plus retrouvées. Les traumatismes cranio-encéphaliques (TCE) dans 58,0 % des cas étaient les affections neurochirurgicales les plus fréquentes.

## Conclusion

Les affections neurochirurgicales constituent un problème majeur de santé publique et sont dominées par les traumatismes cranio-encéphaliques à l'Hôpital ANAIM de Kamsar.

# Research article: Hamartome angiomateux eccrine chez un nourrisson

**Published:** 2023-06-29

**Authors:** Mamadou Diouldé KANTÉ, Kade FOFANA, Mariama Bobo BAH, Marguerite Bomboh BANGOURA, Aissatou Bobo DIALLO, Fanta KABA, Thierno Mamadou TOUNKARA

**Affiliations:** 1 Service de dermatologie, Hôpital national Donka, CHU de Conakry, Guinée; 2 Faculté des sciences et techniques de la santé, Université Gamal Abdel Nasser de Conakry, Guinée

## Contexte

L'hamartome angio-eccrine (HAE) est une dermatose tumorale bénigne, caractérisée par une prolifération de glandes eccrines et de structures vasculaires du derme moyen. Nous rapportons un cas d'HAE remarquable par la rareté et la présentation clinique particulière chez un nourrisson.

## Observation

Il s'agissait d'un nourrisson âgé de 14 mois, né à terme, sans antécédents pathologiques particuliers, reçu en consultation pour une plaque asymptomatique acquise localisée à la cuisse droite, évoluant depuis 3 mois. Son statut vaccinal était à jour. L'examen physique mettait en évidence une plaque érythémateuse, arrondie, à surface lisse, brunâtre, solitaire, mesurant 8 cm de grand axe, siégeant à la face antérieure de la cuisse droite. L'aspect clinique faisait évoquer une tumeur de Spitz. La biopsie cutanée avec examen histopathologique mettait en évidence une prolifération de structures ductuales eccrines associées à des structures vasculaires capillaires et une composante adipeuse, le tout au sein d'un stroma myxoide. Cet aspect était celui d'un HAE. Au décours d'un suivi de 12 mois sans traitement, la lésion est restée stable.

## Discussion

C'est une pathologie rare dont l'incidence est inconnue. Il s'agit le plus souvent de cas cliniques ou de séries ouvertes. L'HAE survient le plus souvent à la naissance comme rapporté dans notre observation. Ses présentations cliniques sont variables. Elles sont le plus souvent à type de plaque érythémateuse ou **érythémato-téla**ngiecta-sique. L'HAE se situe généralement dans le derme profond, contenant un nombre accru de structures eccrines et de nombreux canaux capillaires entourant ou entremêlés avec les structures eccrines. Sur le plan thérapeutique, l'ablation chirurgicale reste le traitement de référence pour les patients qui présentent des lésions symptomatiques (invalidantes), d'hypertrophie progressive ou de sensibilité esthétique.

# Research article: Acceptation et perception sur la vaccination anti-Covid-19 au sein de la population générale de la commune de Dixinn, Conakry, en 2021

**Published:** 2023-06-29

**Authors:** Niouma Nestor LENO, Foromo GUILAVOGUI, Dana CAMARA, Alioune CAMARA, Alexandre DELAMOU

**Affiliations:** 1 Faculté des sciences et techniques de la santé, Université Gamal Abdel Nasser de Conakry, Guinée; 2 Centre d'excellence africain de prévention et de contrôle des maladies transmissibles, Conakry, Guinée; 3 Programme national de lutte contre le VIH/sida et les hépatites, Guinée

## Introduction

Cette étude visait à évaluer l'acceptation et décrire les perceptions des habitants de la commune de Dixinn sur le vaccin anti-Covid-19.

## Méthodes

Étude transversale à visée analytique d'une durée de six (6) mois allant du 1^er^ juin au 30 novembre 2021 réalisée auprès de 972 habitants de la commune de Dixinn. Un échantillonnage en grappe à trois degrés a été utilisé pour la sélection des cibles (secteurs, ménages et participants). Les variables quantitatives ont été résumées sous forme de médiane avec intervalles interquartiles. Les variables catégorielles ont été résumées sous forme de proportion avec intervalles de confiance. Une régression logistique multivariée a été utilisée pour étudier les facteurs associés à la non-acceptation de la vaccination anti-Covid-19 (p ≥ 0,05).

## Résultats

L’âge moyen des participants était de 26,29 ans (15-26). Il a été noté dans cette étude que 73,1 % des participants accepteraient de se faire vacciner avec les vaccins proposés par le gouvernement guinéen. Les facteurs associés à la non-acceptation du vaccin anti-Covid-19 étaient la faible connaissance sur la maladie coronavirus (ORa = 3,21; IC95% [2,42-4,75]), la préférence à l'utilisation des plantes médicinales pour le traitement de la Covid-19 (ORa = 1,59; IC95 % [1,08-2,33]), le statut élève/étudiant (ORa = 1,30; IC95% [0,42-3,90]) et le statut de profession libérale (ORa = 1,14; IC95% [0,40-1,84]). Les principaux motifs de refus de la vaccination anti-Covid-19 étaient la perception que le vaccin réduirait la reproduction/humanité, les effets secondaires du vaccin et la préférence de laisser la nature faire son cours.

## Conclusion

Cette étude a montré que le taux d'acceptation de la vaccination anti-Covid-19 est globalement bon au sein de la population de Dixinn. Il n'y a donc pas de réel problème de réticence à se faire vacciner contre la Covid-19. Le renforcement des stratégies d'information sur la Covid-19 et les vaccins anti-Covid-19 réduirait les quelques refus de se faire vacciner qui ont été rapportés dans cette étude.

# Research article: Expérience d'utilisation du dolutégravir chez les patients infectés par le VIH-1 à Abidjan de 2017 à 2019

**Published:** 2023-06-29

**Authors:** Kelety CAMARA, Wardatou Dine MOURTADA, N'douba Alain Charles KASSI, Adama DOUMBIA, Ahuatchi Patrick Justin COFFIE, Aminata SYLLA, Fodé Bangaly SAKO, Mamadou Saliou SOW, Serge Paul EHOLIÉ

**Affiliations:** 1 Service des maladies infectieuses et tropicales, Centre hospitalier universitaire de Treichville, Abidjan, Côte d'Ivoire; 2 Service des maladies infectieuses et tropicales, Hôpital national Donka, Conakry, Guinée

## Introduction

Le dolutégravir a présenté une efficacité et une barrière génétique plus élevée ainsi qu'une bonne tolérance. L'objectif était de rapporter l'expérience de l'utilisation du dolutégravir (DTG) chez les patients infectés par le VIH-1.

## Matériel et méthodes

Il s'agit d'une étude rétrospective des dossiers de patients infectés par le VIH, naïfs et prétraités recevant une trithérapie antirétrovirale comportant le dolutégravir, suivis au SMIT entre 2017 et 2019. L'analyse a porté sur les aspects clinico-biologiques, thérapeutiques et évolutifs. L'efficacité et la tolérance du traitement ont été évaluées à partir de M6-M24.

## Résultats

Les dossiers de 331 patients dont 191 femmes (57,70 %), sex-ratio H/F de 0,73, et âge médian de 45 ans ont été retenus. À l'inclusion 119 (35,95 %) naïfs de traitement antirétroviral et 212 (64,05 %) prétraités, parmi lesquels 85 (40,09 %) étaient en échec virologique. À M24 du suivi, la charge virale était supprimée chez tous les naïfs et dans la majorité des cas (97,17 %) chez les prétraités. Concernant la tolérance, aucun effet indésirable grave n'a été notifié. Les effets secondaires les plus rapportés étaient les troubles neuropsychiatriques chez 80,36 % des naïfs contre 70,69 % des prétraités. Une augmentation de l'IMC médian à M24 était observée, de 18,93 kg/m^2^ à 23 kg/m^2^.

## Conclusion

Cette étude rapporte une efficacité et une tolérance optimales du dolutégravir chez les personnes vivant avec le VIH.

# Research article: Prise en charge des femmes séropositives au VIH ayant contracté une grossesse au cours du traitement antirétroviral

**Published:** 2023-06-29

**Authors:** Abdoulaye Mamadou TRAORÉ, Amadou BAH, Aïssata DIALLO, Garan DABO, Hamsetou CISSÉ, Tiounkani THERA, Daouda K. MINTA

**Affiliations:** CHU Pr Bocar Sidi Sall, Kati, région de Koulikoro, Mali

## Introduction

L'association grossesse et VIH constitue une situation à haut risque de complications materno-fœtales et de transmission du VIH à l'enfant en l'absence de mesures idoines. Dans le but d’évaluer nos pratiques en matière de prévention de la transmission mère-enfant (PTME), nous avons initié cette étude afin de déterminer la fréquence des grossesses au cours du suivi, le pronostic maternel et fœtal et la prévalence de la séroconversion au VIH du nouveau-né, voire de l'enfant.

## Méthodologie

Il s'agit d'une étude de cohorte rétrospective incluant les dossiers des femmes infectées par le VIH suivies entre le 1^er^ janvier et le 31 décembre 2018 au CHU Pr Bocar Sidi Sall de Kati (région de Koulikoro, Mali).

## Résultats

Sur un total de 996 PvVIH constituant la cohorte, 480 sont de sexe féminin (48,19 %). Parmi elles, 52 avaient contracté une grossesse (10,8 %) dont 30 pour la période indiquée. Avec un âge moyen de 30 ans ± 14 ans, 60 % étaient paucigestes, 30 % multigestes et 10 % primigestes. La charge virale réalisée chez 28/30 était indétectable (21/30; 70 %) et supérieure à 1000 copies/ml chez 7 (23,33 %). Le taux de CD4 était inférieur à 350/mm^3^ chez 5 cas. Toutes étaient soumises au traitement antirétroviral (TARV) dont 63,34 % en 1^re^ ligne et 36,66 % sous schéma de 2^de^ ligne. La grossesse est arrivée à terme (27; 90 %) et 3 ont accouché prématurément. À l'issue, 29 nouveau-nés ont survécu (dont 2 cas de petit poids à la naissance) contre 1 mortalité néonatale. À 2 ans de vie, 3 nourrissons (10,34 %) ont été infectés par le VIH et 1 était décédé.

## Conclusion

Malgré la mise sous TARV, certaines femmes contractent une grossesse en l'absence de la suppression virologique. Le taux de transmission du VIH de la mère à l'enfant reste non négligeable. Il convient d'approfondir les recherches pour identifier les facteurs et formuler des recommandations en vue de l’élimination de la transmission mère-enfant du VIH à Kati.

# Research article: Profil lipidique des patients malades rénaux chroniques au Service de néphrologie du CHU Donka

**Published:** 2023-06-29

**Authors:** Mouhamadou Madiou BAH, Mamadou Saliou BALDE, Thierno WANN, Alpha Oumar BAH

**Affiliations:** 1 Service de néphrologie-hémodialyse, Hôpital national Donka, CHU de Conakry, Guinée; 2 Service de m**édecine interne,** Hôpital national Donka, CHU de Conakry, Guinée

La maladie rénale chronique (MRC) est associée à une altération du métabolisme des lipoprotéines dans l'organisme qui est à la fois qualitative et quantitative. Les anomalies les plus importantes sont une augmentation des triglycérides, une diminution du cholestérol HDL et une augmentation du cholestérol LDL responsable d'un profil lipidique très athérogène. Notre étude avait pour objectif d’étudier le profil lipidique des patients MRC et d'identifier les autres facteurs de risque cardiovasculaires (FDRCV).

## Matériel et méthodes

Il s'agissait d'une étude d'observation d'une durée de 6 mois allant du 15 avril au 15 septembre 2021 réalisée dans le Service de n**éphrologie du CHU** Donka. L’étude avait concerné l'ensemble des patients atteints de la MRC associée à une dyslipidémie ayant été admis ou hospitalisés dans le service.

## Résultats

Soixante-dix-sept (77) patients ont été retenus, soit une prévalence de 74 %. L’âge moyen était de 49,1 ± 17,7 ans. Outre la dyslipidémie, l'HTA était le principal FDRCV rencontré, suivi de l’âge. La NV était l’étio-logie la plus fréquente. Le profil lipidique des patients atteints de la MRC était représenté par une dyslipidémie mixte dans 80,5 % des cas, caractérisée par une hypertriglycéridémie, une augmentation du cholestérol LDL et une baisse du cholestérol HDL. L'hypertriglycé-ridémie était fréquente, dans 87 % des cas. L'hypercholestérolémie LDL était statistiquement associée à la survenue de décès, de l'artériopathie oblitérante des membres inférieurs (AOMI) et de l'AVC (P_1_ = 0,02; P_2_ = 0. 00; P_3_ = 0,04).

## Conclusion

Le profil lipidique est caractérisé par une dyslipidémie mixte, les complications cardiovasculaires sont fréquentes et responsables de la mortalité.

# Research article: Itinéraire thérapeutique des patients tétaniques admis au Service des maladies infectieuses de l'Hôpital national Donka

**Published:** 2023-06-29

**Authors:** Tamba Michel KAMANO, Kelety CAMARA, Mamadou Oury Safiatou DIALLO, Fodé Bangaly SAKO, Fodé Amara TRAORÉ, Mamadou Saliou SOW

**Affiliations:** 1 Service des maladies infectieuses et tropicales, Hôpital national Donka, CHU Conakry, Guinée; 2 Université Gamal Abdel Nasser de Conakry, Guinée

## Introduction

Malgré l'existence d'un vaccin disponible, le tétanos demeure une maladie mortelle. L'objectif de cette étude était d'identifier l'itinéraire thérapeutique des patients admis pour tétanos.

## Matériel et méthodes

Il s'agissait d'une étude prospective de type descriptif de six (6) mois allant du 1^er^ janvier au 30 juin 2020 au SMIT. L'analyse a porté sur les aspects clinico-thé-rapeutiques et évolutifs.

## Résultats

Sur 203 cas d'hospitalisation durant la période d’étude, 15 cas de tétanos ont été colligés (7,3 %). Le sexe masculin était davantage retrouvé (80 %). La tranche d’âge de 5 à 15 ans était plus représentée. L’âge médian était de 11 ans. Le groupe socioprofessionnel élève/étudiant était plus retrouvé (73,3 %); la commune de Ratoma était la zone la plus touchée (33,3 %); et 26,6 % des patients ont consulté un tradipraticien. Dans notre étude, le tétanos a été méconnu dans 53,3 % des cas pendant la première consultation médicale alors que 66,6 % des prestataires étaient des médecins, et 40 % de ces prestataires venaient de structures privées. Lors de la 2^e^ consultation médicale, le tétanos est toujours resté méconnu dans 33,3 % des cas par les prestataires, médecins pour la plupart. Seulement 6,6 % des patients ont bénéficié de leur première dose de sérum anti-tétanique (SAT) pour les 2 consultations. La totalité des patients avaient un statut vaccinal défectueux, et 79,9 % des victimes avaient une porte d'entrée tégu-mentaire ou traumatique. Le taux de mortalité était de 33,3 % avec une durée moyenne d'hospitalisation de 7 jours.

## Conclusion

L'itinéraire thérapeutique du tétanos reste encore complexe dans la plupart des cas, ce qui contribue à retarder l'orientation des victimes dans le centre spécialisé.

# Research article: Hémolyse soutenue par lévofloxacine chez un drépanocytaire SS

**Published:** 2023-06-29

**Authors:** Mamadou Cellou BALDE, Mamadou Saliou BALDE, Alpha Boubacar BAH, Ibrahim CHERIF, Luciana SPATARU, Zeinab AWADA, Laetitia CAUMETTE, Marie-Annick CADEAC, Bernard DELMAS, Philippe MONTANE DE LA ROQUE, Joséphine THOMAZEAU, Algassimou BAH, Mamadou Sere BAH, Mohamed Lamine KABA, Alpha Oumar BAH

**Affiliations:** 1 Centre hospitalier intercommunal des Vallées de l'Ariège, Foix, France; 2 Service de néphrologie-hémodialyse, Hôpital national Donka, Conakry, Guinée; 3 Service de médecine polyvalente, Centre hospitalier de Longjumeau, France

La survenue d'une hémolyse aiguë chez un patient ayant une hémoglobinose stable peut être d'origine corpusculaire ou extra-corpusculaire.

## Observation du cas

Nous rapportons le cas d'un patient drépanocytose homozygote (SS) stable depuis une vingtaine d'années, qui est traité par lévofloxacine pour une septicémie à point de départ urinaire. La biologie : une hyperleucocytose à 33 570/mm^3^ faite de polynucléaires neutrophiles à 90 % et une CRP à 160 mg/l, une anémie hémolytique, mécanique, microcytaire non thrombopénique (Hb à 8,3 g/dl), une haptoglobuline effondrée < 0,1, des schizocytes faiblement positifs < 0,1 %; des PL à 277 000 sans argument pour un CIVD, le taux du fibrinogène est plutôt élevé à 5,68 g/l et l'hémostase est normale. Les protides sont contractés à 93 g/l. Le bilan hépatique montre une bilirubine T à 144 Umol/l, bilirubine conjuguée à 38 Umol/l, bilirubine libre à 106 Umol/l, PAL à 166, ASAT et ALAT à 50 Umol/l, Gamma GT et amylase sont normales. Le bilan martial est plutôt inflammatoire avec un Fer sérique bas à 8 μmol/l, une saturation à 13 % et une ferritinémie élevée à 462 μg/l. L'ECBU revient positif à *Enterobacter chloacae* avec une leucocyturie significative à plus de 100 000/ml compliquée d'une bactériémie au même germe.

## Discussion

La présente observation attire l'attention sur la possibilité d'entretenir une hémolyse intra-vasculaire par des agents infectieux et pharmacologiques. Alors que l'infection semblait être maîtrisée et que le patient devenait apyrétique, il continuait à présenter des signes d'hémolyse suspectée comme étant secondaire à la poursuite du traitement par la lévofloxacine. Cette dernière a été décrite comme induisant une hémolyse par un mécanisme auto-immun ou secondaire à un déficit en G6PD – mécanisme commun avec tous les fluoroquinolones.

## Conclusion

La lévofloxacine vient démasquer un déficit en G6PD à l'origine d'une hémolyse chez un patient drépanocytaire homozygote.

# Research article: Covid-19 : profils épidémiologiques, cliniques et pronostics des sujets âgés en réanimation au CT-ÉPI de Gbessia

**Published:** 2023-06-29

**Authors:** M'mah Lamine CAMARA, Joseph DONAMOU, Thierno Saidou DIALLO, Hans HOUNMENOU

**Affiliations:** 1 Service d'anesthésie-réanimation, Hôpital national Donka, Conakry, Guinée; 2 Université Gamal Abdel Nasser de Conakry, Guinée

## Objectif

Décrire les caractéristiques épidémiologiques cliniques et évolutives des sujets âgés admis en réanimation au CT-ÉPI de Gbessia.

## Matériel et méthodes

Il s'agissait d'une étude prospective observationnelle de type descriptif, transversale, d'une durée de 9 mois allant de décembre 2020 à août 2021.

## Résultats

Durant notre étude, 665 patients ont été admis en réanimation avec 360 d'entre eux qui avaient un âge supérieur à 65 ans, soit une prévalence de 54,1 %. L’âge moyen des patients était de 74,5 ± 7,4 ans. Le sexe masculin était le plus représenté soit 62,5 %. L'HTA était la comorbidité la plus collectée soit 31,4 % des cas, suivie de l'HTA + diabète avec 25,3 % des cas. La symptomatologie était représentée par asthénie physique (98,9 %), toux (90,3 %), trouble de la conscience (83,1 %), dyspnée (77,2 %), anorexie (73,6 %), céphalées (73,6 %) et nausée (59,7 %). Le syndrome de détresse respiratoire aiguë était la complication la plus représentée avec 41,4 %. Le taux de létalité était de 14,7 % sur l'ensemble des patients. La durée moyenne d'hospitalisation était de 7,7 jours.

## Conclusion

Le sujet âgé représente un peu plus de la moitié des patients admis en réanimation. Ils étaient pour la plupart de sexe masculin et avaient au moins une comorbidité. Leur symptomatologie était dominée par l'asthénie physique, la toux et la dyspnée.

# Research article: Les infections opportunistes du système nerveux au cours du sida : aspects épidémiologiques, diagnostiques et évolutifs

**Published:** 2023-06-29

**Authors:** Mamoudou SAVADOGO, Ismael DIALLO, Kognimisson Apoline SONDO, Zoumbahan Marie Thérèse TRAORÉ

**Affiliations:** 1 Service des maladies infectieuses, CHU Yalgado Ouédraogo, Ouagadougou, Burkina Faso; 2 Service de médecine interne et Service des maladies infectieuses, CHU Yalgado Ouédraogo, Ouagadougou, Burkina Faso

## Introduction

Les infections opportunistes à localisation neurologique sont responsables d'une lourde létalité des PvVIH. L'objectif de notre étude est de décrire les aspects épidémiologiques, diagnostiques et évolutifs des infections opportunistes à localisation neurologique dans le Service des maladies infectieuses en 2019.

## Patients et méthodes

Il s'est agi d'une étude rétrospective couvrant la période du 1^er^ janvier au 31 décembre 2019. Étaient inclus tous les patients admis et hospitalisés pour infection opportuniste à localisation neurologique à partir des arguments cliniques, biologiques et/ou tomodensitométriques.

## Résultats

Durant la période d’étude, 159 patients avaient été hospitalisés dans le Service des maladies infectieuses. Parmi eux 10 cas de toxoplasmose cérébrale, 3 cas de cryptococcose neuroméningée et 1 cas de tuberculose méningée avaient été diagnostiqués, soit une fréquence hospitalière de 6,28 %, 2 % et 0,6 % respectivement. L’âge moyen des patients était de 40 ans ± 9. Soixante et un pour cent (61 %) des patients étaient de sexe féminin contre 39 % de sexe masculin soit un sexratio de 0,64. La majorité des patients (80 %) provenait de la capitale Ouagadougou et de ses banlieues. Les infections opportunistes sont survenues chez des patients infectés par le VIH sévèrement immunodéprimés (taux de lymphocytes TCD4 moyen 59 ± 13 cell/mm^3^). Les motifs d'hospitalisation étaient dominés par une altération de l’état général, une difficulté à la marche et à l’élocution, une fièvre et des céphalées. L'examen clinique notait un déficit moteur et un syndrome méningé chez la majorité des patients. La tomodensi-tométrie avait contribué au diagnostic chez tous les patients souffrant de toxoplasmose cérébrale. Le diagnostic de la cryptococcose neuroméningée a été posé à partir de l'examen du liquide cérébrospinal (LCS) à l'encre de Chine. Quant à la tuberculose méningée, son diagnostic a été retenu à partir de l'examen cytobactériologique et biochimique du LCS. La toxoplasmose cérébrale a été la circonstance du dépistage de l'infection à VIH chez 40 % des patients, tandis que la cryptococcose neuroméningée l'a été chez 23 %. La toxoplasmose et la cryptococcose neuroméningée sont survenues chez respectivement 60 % et 72 % des patients en échec du traitement antirétroviral. Sous traitement, l’évolution a été marquée par une létalité de 22,5 %.

## Conclusion

Le terrain de prédilection des infections opportunistes du système nerveux demeure les patients infectés par le VIH sévèrement immunodéprimés naïfs du traitement antirétroviral ou en échec thérapeutique.

# Research article: Maladie rénale et infection à VIH : aspects épidémiologique et clinique à l'Hôpital régional de N'Zérékoré (Guinée)

**Published:** 2023-06-29

**Authors:** Alpha Boubacar BAH, Mamadou Saliou BAH, Fouceny DIAKITÉ, N. MEKOYO, Ibrahima CHERIF, Moussa TRAORÉ, S. BANGOURA, Mohamed Lamine KABA, Alpha Oumar BAH

**Affiliations:** Service de néphrologie-hémodialyse, Hôpital national Donka, Conakry, Guinée

## Introduction

La maladie rénale chez les personnes infectées par le VIH se manifeste de diverses manières, y compris l'insuffisance rénale aiguë (IRA), l'insuffisance rénale associée au VIH, l'insuffisance rénale chronique (IRC) et la toxicité rénale liée au traitement. La prévalence des anomalies rénales a été estimée à environ 30 % des patients. Il s'agissait d'une étude transversale prospective à visée descriptive sur une période de six (6) mois allant du 30 avril au 30 octobre 2017. Les objectifs de notre étude étaient d’évaluer les aspects épidémiologique et clinique.

## Discussion

Durant notre étude, 147 patients infectés par le VIH ont été enregistrés dans le Service de médecine générale de l'Hôpital régional de N'Zérékoré, parmi lesquels 62 (42,18 %) ont répondu à nos critères et présenté la maladie rénale. La tranche d’âge de 46-55 ans était la plus représentée (48,39 %). La quasi-totalité de nos patients avaient de l'asthénie physique (91,94 %), suivie de la fièvre (83,87 %) et des céphalées (80,65 %). Les signes physiques les plus présents étaient la pâleur des téguments et conjonctives (56,45 %) et les œdèmes des membres inférieurs (43,55 %).

Les marqueurs d'atteinte rénale étaient dominés par la protéinurie avec 69,35 %. Sur les 62 patients présentant une maladie rénale, 47 (75,80 %) avaient des atteintes rénales organiques et les types les plus représentés étaient les atteintes glomérulaires avec 43,55 %, suivies des atteintes tubulo-interstitielles avec 22,58 %. L'insuffisance rénale chronique était chez 60 % contre 40 % des cas pour l'insuffisance rénale aiguë.

## Conclusion

La maladie rénale est très fréquente et variée au cours du VIH.

# Research article: Investigation ethnomédicale et évaluation de l'activité antiplasmodiale des Terminalia spp de Guinée

**Published:** 2023-06-29

**Authors:** Alpha Oumar BALDE, Fatoumata BAH, Alimou CAMARA, Aicha DRAME, Ferima DEMBELE, Mamadou Aliou BALDE, Mohamed Sahar TRAORÉ, Elhadj Saidou BALDE, Sere DIANE, Mamadou Billo BAH, Thierno Dardaye BAH, Aliou Mamadou BALDE

**Affiliations:** 1 Université Gamal Abdel Nasser de Conakry, Conakry, Guinée; 2 Institut de recherche et de développement des plantes médicinales et alimentaires de Guinée (IRDPMAG), Conakry, Guinée

## Introduction

En Guinée le paludisme affecte 44 % de la population (INS, 2017) et le recours aux plantes médicinales dans la résolution des problèmes de santé est une pratique très ancienne. Les plantes du genre *Terminalia* sont d'usage traditionnel dans le traitement de plusieurs affections. Notre objectif est de faire une investigation ethnomédicale de six espèces de *Terminalia* rencontrées en Guinée dans la prise en charge du paludisme.

## Méthodologie

Une enquête ethnobotanique suivie de récolte a été réalisée dans les préfectures de Dubréka, Labé, Kankan et Lola. Des extraits polaires et apolaires des espèces récoltées ont été préparés et des tests antiplas-modial et cytotoxique ont été réalisés.

## Résultats

Au total, 100 personnes ont été interviewées, parmi lesquelles 79 tradithéra-peutes et 21 herboristes qui connaissaient au moins une espèce de *Terminalia*. S'agissant du mode d'acquisition du savoir : (52/100) l'ont obtenu par héritage, (27/100) par apprentissage, (18/100) par expérience personnelle et (3/100) par guérison d'une maladie. Usages des plantes : *T. albida* (91/100), *T. macroptera* (48/100), *T. glaucescens* (24/100), *T. ivorensis* (7/100), *T. catappa* (8/100). Usages thérapeutiques : paludisme (37/100), hémorroïdes (45/100), dermatoses (25/100), rhume (22/100.) Des extraits préparés, il en ressort les résultats suivants :

Activité antiplasmodiale prononcée (IC_50_ ≤ 5 μg/ml) : *T. glaucescens, T. macroptera, T. ivorensis* (tige et racine), *T. superba* (racine); bonne (IC_50_ ≤ 10 μg/ml) : *T. albida* (racine), *T. catappa* (racine et tige). Tous les extraits ont été cytotoxiques sauf l'extrait méthanolique de tige de *T. superba* (CC_50_ = 33,45 μg/ml). Cytotoxicité élevée : extrait de racine de *T. ivorensis* (CC_50_=1 μg/ml). Cytotoxicité modérée : racine et tige *T. glaucescens, T. catappa, T. macroptera, T. albida, T. superba,* et tige de *T. ivorensis.*

## Conclusion

*Terminalia spp* ont montré un effet antipaludique significatif *in vitro* qui corrobore l'usage traditionnel. Les recherches sont en cours pour le fractionnement bioguidé et l'isolement des principes actifs.

# Research article: Charge virale de rattrapage chez les patients sous traitement antirétroviral au Service des maladies infectieuses et tropicales de l'Hôpital national Donka

**Published:** 2023-06-29

**Authors:** Aguibou SOW, Mamadou Alimou TOURÉ, Kelety CAMARA, Ibrahima BAH, Mamadou Oury Safiatou DIALLO, Thierno Mamadou TOUNKARA, Fodé Amara TRAORÉ, Fodé Bangaly SAKO, Mamadou Saliou SOW

**Affiliations:** Service des maladies infectieuses et tropicales, Hôpital national Donka/Université Gamal Abdel Nasser de Conakry, Guinée

## Introduction

La charge virale de rattrapage consiste à tester tous les patients ayant manqué la réalisation de la charge virale au moment du suivi sous traitement antirétroviral (TARV) et sans tenir compte du temps passé sous traitement. L'objectif de cette étude était d’évaluer le suivi virologique des patients sous TARV au Service des maladies infectieuses et tropicales (SMIT) de l'Hôpital national Donka.

## Matériel et méthodes

Il s'agit d'une étude transversale observationnelle et analytique portant sur les patients infectés par le VIH-1 sous traitement ARV suivis au SMIT au moins depuis 6 mois portant sur la période du 1^er^ mars au 31 mai 2020, soit une durée de 3 mois. L'analyse a porté sur les aspects clinico-biologiques, thérapeutiques et évolutifs.

## Résultats

Sur un total de 1605 patients suivis durant la période au SMIT de Donka, 875 avaient manqué la réalisation de la charge virale au moment du suivi à 6 mois sous TARV, soit une proportion de 54,52 %. Nous avons noté une prédominance féminine à 72,30 % avec un âge médian de 39,62 ± 10,81 ans [18 à 80 ans].

Les raisons de manquement du suivi de la charge étaient la demande non faite par les médecins dans 90,4 % des cas et le non-respect du rendez-vous par le patient dans 9,6 %.

Nous n'avons pas trouvé de facteurs statistiquement associés au manquement du suivi de la charge virale.

## Conclusion

Il ressort de notre étude que la proportion de patients ayant manqué la charge virale de suivi est élevée. Ces résultats traduisent une négligence des prestataires dans le suivi biologique des patients sous trithérapie antirétrovirale.

# Research article: Corrélation entre le compte rendu du scanner thoracique et le résultat du test RT-PCR dans le diagnostic positif de la Covid-19 à Conakry

**Published:** 2023-06-29

**Authors:** Oumou Hawa DIALLO, Demba TOURÉ, Alpha Oumar BARRY, Thierno Hassan DIALLO, Aboubacar CAMARA, Alpha Oumar KANTÉ, Boubacar Djelo DIALLO, Lansana Mamadi CAMARA

**Affiliations:** 1 Faculté des sciences et techniques de la santé, Université Gamal Abdel Nasser de Conakry, Guinée; 2 Service de pneumologie, Hôpital national Ignace Deen, Conakry, Guinée

## Contexte

La Covid-19 est une maladie due à un virus zoonotique appelé SARS-CoV-2 (le coronavirus 2 du syndrome respiratoire aigu sévère), apparu en décembre 2019. Elle a provoqué l'une des plus grandes épidémies mondiales au cours de ces dernières années. Le but de cette étude est d’étudier la valeur diagnostique et la cohérence du scanner thoracique par rapport au test RT-PCR dans la prise en charge de la Covid-19 à Conakry.

## Patients et méthodes

Le Centre de diagnostic de la Caisse nationale de sécurité sociale a servi de cadre. Il s'agissait d'une étude transversale, analytique d'une durée de 6 mois (1^er^ novembre 2020 au 30 avril 2021). Ont été inclus tous les cas suspects et confirmés de Covid-19, chez lesquels un scanner thoracique et un test de RT-PCR ont été réalisés. Le test khi-deux et une p-value inférieure à 0,05 ont été utilisés pour l'analyse statistique.

## Résultats

Au total, 193 patients suspects de Covid-19 ayant bénéficié d'un scanner thoracique, parmi lesquels 122 (63,2 %) avaient également une RT-PCR. Le scanner thoracique était évocateur d'une pneumonie à SARS-CoV-2 chez 107, et 85 avaient une RT-PCR positive. Ils étaient majoritairement des hommes (73,8 %) avec un sex-ratio de 2,81. L’âge moyen était de 54,08 ±14,02 ans et la majorité résidaient dans la commune de Ratoma (36,1 %). La toux, la fièvre et la dyspnée étaient les symptômes les plus fréquents, respectivement 71,3 %, 67,2 % et 40,2 %. Le scanner thoracique a montré des plages d'opacité en verre dépoli dans 86,1 % des cas. La RT-PCR était positive chez 85 patients soit 69,7 %. La durée entre le prélèvement et le rendu des résultats de la RT-PCR était de 48 h chez 58,2 %. L'analyse statistique bi-variée entre la plus grande majorité des résultats de la RT-PCR et ceux du scanner n’était pas significative avec des p-values qui étaient supérieures à 0,05 à l'exception de celle entre les résultats de la RT-PCR et l'un des résultats du scanner (lésions nodulaires réticulaires/ épaississement des septaux inter-lobulaires) qui était significative, avec une p-value de 0,006. Ce qui nous permet de déduire qu'il y a une corrélation entre les résultats de la RT-PCR et ces types de lésions visibles au scanner.

## Conclusion

La Covid-19 étant une pathologie respiratoire, les cliniciens pourraient utiliser le scanner pour diagnostiquer rapidement les personnes suspectées de la maladie et envisager un traitement avant les résultats de la RT-PCR qui nécessite un équipement spécialisé et prend au moins 24 h, ou lorsqu'un test RT-PCR est négatif.

# Research article: Séroprévalence et facteurs associés au portage de l'AgHBs en milieu carcéral guinéen

**Published:** 2023-06-29

**Authors:** Mamadou Saliou SOW, Mamadou Oury Safiatou DIALLO, Ibrahima BAH, Saidouba Cherif CAMARA, Ibrahima CAMARA, Édouard Florent BANGOURA, Moussa BALDÉ, Dembo DIAKITÉ, Lansana CAMARA, Ansoumane CAMARA, Fodé Bangaly SAKO, Fodé Amara TRAORÉ

**Affiliations:** 1 Service des maladies infectieuses et tropicales, Hôpital national Donka, Conakry, Guinée; 2 Centre de recherche et de formation en infectiologie de Guinée (CERFIG), Conakry, Guinée

## Objectif

Déterminer la prévalence et les facteurs associés au portage de l'AgHBs en milieu carcéral guinéen.

## Matériel et méthodes

Étude observationnelle transversale, multicentrique descriptive et analytique qui s'est déroulée sur une période de six mois dans les maisons centrales des différentes régions administratives de la Guinée. Nous avons inclus les détenus de tout âge, de tout sexe ayant accepté de participer à l’étude et chez qui le dépistage de l'hépatite virale B a été réalisé. Les informations sociodémo-graphiques, cliniques et biologiques ont été collectées.

L'AgHBs a été recherché sur les prélèvements de sang veineux grâce au test rapide CYPRESS AgHBs BANDELETTES^®^. Les facteurs associés au portage de l'AgHBs ont été analysés à l'aide d'une régression logistique multivariée.

## Résultats

Sur 873 détenus, 153 soit une prévalence de 17,5 IC95% [15,1-19,8] étaient porteur de l'AgHBs. L’âge moyen a été de 29,70 ± 10,08 ans, le sexe masculin 96,9 %. Les proportions de l'AgHBs étaient respectivement de 21,7 et 20,5 % à Boké et Kindia. L'analyse logistique multivariée a révélé que la durée de détention de 5-10 ans (OR = 2,20, IC95% [1,05-4,63], p = 0,03), de plus de 10 ans (OR = 4,08, IC95% [1,49-11,18], p < 0,01), le partage de lame entre détenus (OR = 4,08, IC95% [2,07-4,38], p < 0,01), l'usage de cocaïne (OR= 7,75, IC95% [1,66-36,09], p < 0,01) étaient indépendamment associés au portage de l'AgHBs.

## Conclusion

La prévalence de l'infection à VHB parmi les détenus reste élevée. Les facteurs indépendamment associés au portage de l'AgHBs ont été les détentions de plus de 5 ans, le partage de lame et l'usage de cocaïne. La lutte contre cette affection en milieu pénitentiaire nécessite un dépistage, une sensibilisation et une réforme de l'offre des soins. Des études plus approfondies sur la circulation virale et l'impact du milieu carcéral sur l'infection par le VHB semblent nécessaires.

# Research article: La maladie de Behçet : à propos d'une observation au Service de médecine interne à l'Hôpital national Donka

**Published:** 2023-06-29

**Authors:** Thierno Amadou WANN, Abdoul Karim BALDE, Mamadou Lamine Yaya BAH, Amadou KAKE, Djibril SYLLA

**Affiliations:** 1 Service de médecine interne, Hôpital national Donka, CHU de Conakry, Université de Conakry, Guinée; 2 Service d'ophtalmologie, Hôpital national Donka, CHU de Conakry, Université de Conakry, Guinée; 3 Service de diabétologie-endocrinologie et maladies métaboliques, Hôpital national Donka, CHU de Conakry, Université de Conakry, Guinée

## Contexte

La maladie de Behçet (MB) est peu rapportée en Afrique, probablement du fait d'une méconnaissance de ses différents modes de présentation et d'un préjugé sur sa rareté.

## Observation

Il s'agit d'un garçon de 18 ans vu en consultation pour une baisse de l'acuité visuelle avec une uvéite associée à une aphtose buccale et génitale récidivante. Il avait un syndrome inflammatoire biologique non spécifique. Le test pathergique et le HLA B-51 n'ont pas été réalisés. Le diagnostic de maladie de Behçet est retenu devant l'atteinte tripolaire. Il a été mis sous corticoïde (prednisone) et sous colchicine. L’évolution a été favorable dès 3 mois de traitement avec amélioration de sa vision ainsi que la disparition des aphtoses.

## Commentaires

La MB est une vascularite systémique qui peut intéresser tout le corps. Son étiologie n'est pas connue à ce jour, mais des facteurs génétiques et environnementaux sont incriminés. Son diagnostic est essentiellement clinique. C'est une affection chronique qui est beaucoup plus retrouvée sur l'ancienne « route de la soie » incluant des pays comme la Turquie et l'Iran. Cependant, les brassages entre peuples ont certainement contribué à sa répartition dans le monde. Elle est retrouvée de plus en plus en Afrique subsaharienne. De plus, l'expression HLA B-51 n'est pas fréquente en cas de MB chez le sujet de pigmentation foncée.

## Conclusion

La MB doit être évoquée devant la triade « uvéite, aphtose buccale, aphtose génitale » et en cas de manifestations systémiques. Le préjugé sur sa rareté peut entraîner un retard diagnostique en Afrique.

# Research article: Caractéristiques et facteurs associés au décès dans le cadre d'une hospitalisation pour diarrhée au cours de l'infection à VIH

**Published:** 2023-06-29

**Authors:** Aly MANSARÉ, Daye KA, N. LO, K. SALL, Mbaye Khardiatou DIALLO, Ndeye Aissatou LAKHE, Viviane Marie Pierre CISSÉ DIALLO, Cheikh Tidiane NDOUR, Moussa SEYDI

**Affiliations:** 1 Service des maladies infectieuses, Centre hospitalier national universitaire de Fann, Dakar, Sénégal; 2 Université Cheick Anta Diop, Dakar, Sénégal

## Objectifs

Déterminer la prévalence de la diarrhée chez les PvVIH; Décrire les aspects épidémiologiques, cliniques, paracliniques, thérapeutiques et évolutifs de la diarrhée chez les PvVIH; Identifier les facteurs associés au décès.

## Matériel et méthodes

Il s'agissait d'une étude transversale, descriptive et analytique allant de janvier 2012 à décembre 2016. Elle a porté sur les dossiers de patients infectés par le VIH hospitalisés pour diarrhée au Service des maladies infectieuses et tropicales du CHNU de Fann.

## Résultats

Durant la période d’étude, la prévalence de la diarrhée chez les PvVIH était de 26,9 %. Le sexe féminin était prédominant avec un sex-ratio de 1,18. L’âge moyen était de 46 ans ± 13. La diarrhée était liquidienne dans 76 % des cas. Le signe d'immunodépression clinique le plus rapporté était la candidose buccale (50 %). La majorité des patients avait une déshydratation sévère (53,5 %). Le taux de LTCD4+ moyen était de 80,7 ± 184 cellules/ mm^3^. La majorité des patients avait une anémie avec un taux moyen d'hémoglobine de 8,4 g/dl ± 2,9. Le *Cryptosporidium parvum* était le parasite le plus rencontré (44,8 %), *Isospora belli* n’était retrouvé que chez 2 patients. La létalité dans cette étude était de 29,7 %. Le nombre de patients ayant pris des anti-rétroviraux était de 54 (37,8 %). En analyse multivariée, seule la non mise sous traitement ARV était significativement associée au décès (p = 0,01).

## Conclusion

L’état des lieux sur la prévalence de la diarrhée chez les PvVIH ainsi que sur les facteurs associés au décès étant fait, d'autres études prospectives multicentriques doivent être réalisées pour mieux cerner les facteurs associés au décès chez les patients infectés par le VIH présentant une diarrhée.

# Research article: Devenir des patients coinfectés VIH/tuberculose suivis au Service des maladies infectieuses et tropicales de l'Hôpital national Donka

**Published:** 2023-06-29

**Authors:** Pélagie WAGUEU, Karamba SYLLA, Aminata SYLLA, Ibrahima BAH, Mamadou Oury Safiatou DIALLO, Fodé Amara TRAORÉ, Fodé Bangaly SAKO, Mamadou Saliou SOW

**Affiliations:** 1 Service des maladies infectieuses et tropicales, Hôpital national Donka, Conakry, Guinée; 2 Faculté des sciences et techniques de la santé, Université Gamal Abdel Nasser de Conakry, Conakry, Guinée

## Introduction

La tuberculose et l'infection à VIH constituent deux problèmes majeurs de santé publique en Afrique subsaharienne. L'objectif de cette étude est d’évaluer le devenir des patients co-infectés par le VIH/TB.

## Matériel et méthodes

Il s'agissait d'une étude transversale prospective portant sur 104 patients co-infectés TB/VIH entre le 1^er^ août 2019 et le 29 février 2020 au Service des maladies infectieuses et tropicales de Donka. Soit une durée de 7 mois.

## Résultats

Les 104 patients suivis étaient majoritairement des femmes (59,6 %), mariés (66,3 %) et avec un âge moyen de 39,75 ± 12,44 ans. Dans notre étude, 80 % des patients avaient un taux de CD4 < 200 cellules/mm^3^ et le stade 4 a été le plus représenté chez nos patients soit 62,5 %. À la fin de l’étude, 45,2 %> étaient guéris contre 29,8 % de décès, 22,2 % de perdus de vue et 2,9 % d'abandons; et le facteur associé au décès était l’âge avec une p-value de 0,033.

## Conclusion

Le couple TB/VIH demeure un problème de santé publique. Il faut donc rechercher le VIH chez tous les patients atteints de tuberculose et rechercher la tuberculose chez les PvVIH.

# Research article: Étude échographique de l'index de protrusion prostatique

**Published:** 2023-06-29

**Authors:** Thierno Hamidou BALDE, Ibrahima Sory DOUMBOUYA, Aminata SACKO, Mamadou KOUROUMA, Amadou DOUMBOUYA

**Affiliations:** 1 Service de radiologie, Hôpital national Ignace Deen, Conakry, Guinée; 2 Service de radiologie, Hôpital national Donka, CHU de Conakry, Guinée; 3 Centre de diagnostic de la Caisse nationale de sécurité sociale, Guinée; 4 Service de radiologie, dCentre de santé de référence de la Commune 6, Bamako, Mali; 5 Service de chirurgie pédiatrique, Hôpital national Donka, CHU de Conakry, Guinée

## Objectifs

Étudier l'apport de l’échographie dans l’évaluation de l'index de protrusion prostatique (IPP) intra-vésicale et déterminer son impact sur la vessie.

## Matériel et méthodes

Il s'agissait d'une étude prospective sur 54 dossiers colligés sur une période de 4 mois, de février à mai 2022. Nous avons inclus tous les patients de tout âge ayant une hypertrophie de la prostate et une protrusion prostatique intra-vésicale. Les examens étaient réalisés à l'aide d'un appareil d’échographie de marque « General Electric ». Les paramètres étaient l’âge, le volume de la prostate, l'index de protrusion prostatique, le résidu post-mictionnel, la paroi de la vessie et les calices rénaux.

## Résultats

Les patients avaient un âge compris entre 50 et 60 ans avec une fréquence de 33,3 %. Nous avons trouvé un volume prostatique compris entre 25 et 50 ml chez 16 patients (29,6 %). L'indice de protrusion prostatique grade 3 était retrouvé chez 26 patients (48,1 %). La paroi de la vessie était épaisse et crénelée (vessie de lutte) chez 46 patients (85,2 %); elle était fine et régulière chez 8 patients (14,8 %). On notait un retentissement rénal chez 14 patients (25 %). Le résidu post-mic-tionnel (RPM) était supérieur à 100 ml chez 21 patients (38,9 %).

## Conclusion

L'IPP est un marqueur permettant d’évaluer le risque de progression clinique de l'obstruction sous-vésicale et d'envisager une attitude thérapeutique.

# Research article: Approche diagnostique à propos de 4 cas d'hémoglobine N-Baltimore au Centre médical et Conseil en santé de Kipé (CEMECO), Conakry (Guinée) dans un pays à ressources limitées et revue de la littérature

**Published:** 2023-06-29

**Authors:** Mamadou Lamine Yaya BAH, Fatoumata Binta DIALLO, Abdoul Goudoussy DIALLO, Mamadou Pathé DIALLO

**Affiliations:** 1 Service de médecine interne, Hôpital national Donka, Université Gamal Abdel Nasser de Conakry, Guinée; 2 Service de pédiatrie, Hôpital national Donka, Université Gamal Abdel Nasser de Conakry, Guinée; 3 Service d'hématologie, Hôpital national Ignace Deen, Université Gamal Abdel Nasser de Conakry, Guinée; 4 Centre médical et Conseil en santé, Kipé, Commune de Ratoma, Conakry, Guinée

## Introduction

L'hémoglobine N-Baltimore est un variant rare d'hémoglobine qui peut être identifié par l'analyse électrophorétique.

## Observations

### Patient 1

Âgé de 13 ans, reçu pour bilan systématique (père porteur d'HbA/HbS et la mère porteuse d'HbA/Hb N-Baltimore). L'examen clinique était normal. Électrophorèse de l'hémoglobine : Hb N- Baltimore =39,4 %; HbS = 26,4 %. Hémogramme : Hb = 11,10 g/ dl; VGM = 75 fl; CCMH = 35 g/dl.

### Patiente 2

Âgée de 9 ans, reçue également pour le même motif, chez qui l'examen clinique était normal. Électrophorèse de l'hémoglobine : Hb N-Baltimore = 39,5 %; HbF = 1,6 %; HbS = 24,2 %. Hémogramme : Hb = 11,70 g/dl; VGM = 71fl; CCMH = 33 g/dl.

### Patiente 3

Âgée de 4 ans, adressée au Centre pour bilan d'une anémie. À l'examen physique : conjonctives hypocolorées, sans ictère. Le reste de l'examen est normal. Électrophorèse de l'hémoglobine : Hb N-Baltimore = 57 %; HbF = 5,3 %; HbS = 34,9 %. Hémogramme : Hb = 9,6 g/dl; VGM = 90 fl; CCMH = 26,1 %.

### Patient 4

Âgé de 34 ans, reçu au Centre pour bilan systématique (découverte de l'Hb N-Baltimore chez sa fille). Examen clinique normal. Électrophorèse de l'hémoglobine : Hb N-Baltimore = 60,2 %; HbF = 1,0 %; HbS = 36 %. Hémogramme : Hb = 11 g/dl; VGM = 89 fl; CCMH = 33 %.

## Discussion

L'hémoglobine N-Baltimore résulte d'une mutation dans le codon 95 de la chaîne β globine, avec remplacement de la lysine par l'acide glutamique qui favorise une mobilité électrophorétique plus rapide, par rapport à l'Hb A. Une étude précédente a montré une interaction de l'Hb N-Baltimore avec l'Hb S chez trois enfants en France. Ce variant a également été décrit en association avec la thalassémie et l'Hb C. Bien que l'Hb N-Baltimore accélère la cristallisation de l'Hb C et contribue aux anomalies de la morphologie des érythrocytes, cette combinaison entraîne une évolution clinique modérée chez les porteurs.

# Research article: Les méningites à *Neisseria meningitidis* au Burkina : revue de la littérature

**Published:** 2023-06-29

**Authors:** Mamoudou SAVADOGO, Ismael DIALLO, Kognimisson Apoline SONDO

**Affiliations:** 1 Service des maladies infectieuses, CHU Yalgado Ouédraogo, Ouagadougou, Burkina Faso; 2 Service de médecine interne et Service des maladies infectieuses, CHU Yalgado Ouédraogo, Ouagadougou, Burkina Faso

## Introduction

Les méningites à *Neisseria meningitidis* constituent une menace permanente au Burkina Faso eu égard à leur potentiel épidémique.

## Objectif

Étudier les aspects épidémiologiques des méningites à *Neisseria meningitidis* au Burkina Faso.

## Patients et méthode

Il s'est agi d'une étude transversale à visée descriptive avec collecte rétrospective des données de la littérature sur les épidémies de méningite à *Neisseria meningitidis* au Burkina Faso.

## Résultats

Le Burkina Faso a subi plusieurs épidémies de méningite à *Neisseria meningitidis* de sérogroupe A. En effet, le pays a été frappé par des épidémies dues à ce sérogroupe au cours des années 1957, 1985, 1994, 1995, 1996, 1997, 2004, 2005, 2006, 2007, 2008 et 2009. L’épidémie de 1996 a été particulièrement meurtrière avec 4363 morts enregistrés. Le sérogroupe W de *Neisseria meningitidis* a été également responsable de plusieurs épidémies dont celles de 1992, 2001, 2002, 2003 et 2012, tandis que le sérogroupe X de *Neisseria meningitidis* **a été responsable de la première grande épidémie en** 2010. Quant au sérogroupe C de *Neisseria meningitidis,* après une première flambée épidémique en 1979, c'est en 2019 que ce sérogroupe a de nouveau été responsable d'une flambée à l'Est du Burkina Faso. Au cours de toutes ces épidémies, les enfants étaient les plus touchés et la létalité était élevée (plus de 14 %). Outre la prise en charge thérapeutique des cas, une riposte vaccinale avait permis de circonscrire ces épidémies. Les vaccins utilisés étaient d'abord des vaccins polysaccharidiques (le vaccin anti-méningococcique A+C, le tétravalent A+C+Y+W), puis depuis 2010, le vaccin conjugué MenAfriVac^®^ qui a permis d’éliminer les épidémies dues au méningocoque (Nm) A. Mais il est noté la persistance de la circulation des autres sérogroupes (W, X, C) de *Neisseria meningitidis.* À la semaine n° 33 des années 2021 et 2022, les données de la surveillance épidémiologique montraient un net recul de *Neisseria meningitidis,* derrière *Streptococcus pneumoniae* qui est devenu la première cause de méningite bactérienne au Burkina Faso.

## Conclusion

La circulation persistante des sérogroupes W, X et C de *Neisseria meningitidis* fait craindre d'autres épidémies de méningite. Il importe de maintenir un niveau élevé de surveillance épidémiologique des méningites à *Neisseria meningitidis* au Burkina Faso.

# Research article: Prévalence et facteurs associés à une déficience visuelle dans la population guinéenne âgée en 2021

**Published:** 2023-06-29

**Authors:** Fremba CAMARA, Thierno Mamadou MILLIMONO, Maxime Dantouma SOVOGUI, Pierre JÉSUS, Alioune CAMARA

**Affiliations:** 1 Chaire d'ophtalmologie, Université Gamal Abdel Nasser de Conakry, Guinée; 2 INSERM U1094, IRD U270, Université de Limoges, CHU Limoges, EpiMaCT - Epidémiologie des maladies chroniques en zone tropicale, Institut d’épidémiologie et de neurologie tropicale, OmegaHealth, Limoges, France; 3 Chaire de santé publique, Université Gamal Abdel Nasser de Conakry, Guinée

## Introduction

La déficience visuelle augmente avec l’âge et serait plus répandue chez les personnes âgées. Cette étude visait à déterminer la prévalence et les facteurs associés à la déficience visuelle chez les personnes âgées en Guinée.

## Méthodes

Cette étude transversale portant sur 1698 sujets âgés de 60 ans et plus a recueilli des données sociodémographiques, cliniques (antécédents médicaux et oculaires notés), anthropométriques et d'acuité visuelle (AV) pour les deux yeux. Cette étude communautaire a utilisé un échantillonnage en plusieurs étapes pour sélectionner les participants des ménages de quatre régions naturelles de la Guinée en 2021. La déficience visuelle a été définie pour tout sujet présentant une AV inférieure à 6/12 (0,5) dans le meilleur œil. Une analyse par régression logistique univariée et multivariée a été réalisée pour identifier les facteurs associés à la déficience visuelle.

## Résultats

Sur les 1698 sujets, l’âge moyen était de 71,6 ± 9,4 ans (extrêmes, 60-115) et 63,5 % étaient des hommes; 38,2 % étaient recrutés en Basse-Guinée et 64,6 % résidaient en zone rurale. La prévalence standardisée selon l’âge et le sexe d'une vision basse et de la cécité était de 24,7 % (intervalle de confiance [IC] à 95 % : 24,5-24,8) et de 11,0 % (IC95% : 11,0-11,1) respectivement. La prévalence normalisée selon l’âge et le sexe de la déficience visuelle était de 35,7 % (IC95% : 35,6-35,8). Les facteurs associés à la déficience visuelle étaient le sexe féminin (rapport de cotes [OR] = 1,28; IC95% 1,00-1,67), avoir 70 ans et plus (2,51; 1,95-3,23), avoir un emploi (1,89; 1,44-2,49), résider en milieu rural (1,96; 1,47-2,61), résider en Basse-Guinée (2,99; 2,06-4,32), résider en Guinée forestière (10,19; 6,45-16,10), résider en Haute-Guinée (9,24; 6,34-13,47), avoir un IMC < 18,5 kg/m^2^ (2,09; 1,38-3,16), avoir un 18,5 kg/m^2^ ≤ IMC <24,9 kg/m^2^ (1,51; 1,122,05), présenter une maladie oculaire (9,91; 6,79-14,47), avoir des troubles de l'activité instrumentale (2,07; 1,33-3,22) et basique (2,27; 1,51-3,39).

## Conclusion

La forte prévalence de la déficience visuelle nécessite une réorganisation des systèmes de santé en Guinée. La prévention, le diagnostic précoce et la prise en charge seront ciblés sur les causes évitables de la déficience visuelle dans la population vulnérable des personnes âgées.
